# Effectiveness of exercise-based rehabilitation for depressive symptoms, anxiety, and health-related quality of life in adults with rheumatoid arthritis: A systematic review and meta-analysis of randomized controlled trials

**DOI:** 10.1371/journal.pone.0352173

**Published:** 2026-07-15

**Authors:** Yan Xiao, Jinghui Zhong, Kebao Zhang, Xuesong Yang

**Affiliations:** 1 Institute of Physical Education and Training, Capital University of Physical Education and Sports, Beijing, China; 2 School of Kinesiology and Health, Capital University of Physical Education and Sports, Beijing, China; 3 School of Recreation and Community Sport, Capital University of Physical Education and Sports, Beijing, China; 4 School of Dance and Martial Arts, Capital University of Physical Education and Sports, Beijing, China; University of Diyala College of Medicine, IRAQ

## Abstract

**Background:**

Rheumatoid arthritis is frequently accompanied by depressive symptoms, anxiety, and impaired health-related quality of life. Although exercise is recommended as part of comprehensive care, its effects on psychological outcomes and quality of life remain uncertain. This systematic review and meta-analysis evaluated structured exercise interventions for adults with rheumatoid arthritis.

**Methods:**

PubMed, Embase, CENTRAL, Web of Science, Scopus, PsycINFO, and EBSCOhost were searched from inception to November 26, 2025. Randomized controlled trials comparing structured exercise with non-exercise control conditions were included. Random-effects meta-analyses used Hedges’g standardized mean differences with 95% confidence intervals. Risk of bias was assessed using the Cochrane Risk of Bias 2 tool, and certainty of evidence was rated using GRADE. The protocol was retrospectively registered with INPLASY (INPLASY202610082).

**Results:**

Fifteen trials involving 1652 participants were included. Compared with non-exercise controls, structured exercise reduced depressive symptoms (11 trials, 685 participants; standardized mean difference, −0.49; 95% confidence interval, −0.70 to −0.28; P < 0.001; I² = 42%; moderate certainty). Low-certainty evidence suggested reductions in anxiety (7 trials, 403 participants; standardized mean difference, −0.45; 95% confidence interval, −0.65 to −0.25; P < 0.001; I² = 0%) and improvements in quality of life or health-related quality of life (7 trials, 564 participants; Hedges’ g standardized mean difference, 0.45; 95% confidence interval, 0.18 to 0.71; P = 0.001; I² = 50%). Subgroup analyses showed no statistically significant difference between conventional exercise and mind-body exercise.

**Conclusions:**

Structured exercise probably reduces depressive symptoms and may reduce anxiety and improve quality of life in adults with rheumatoid arthritis, supporting its use as an adjunct to comprehensive care. Larger, better-reported trials with longer follow-up are needed to clarify durability, clinical importance, and optimal delivery.

## Introduction

Rheumatoid arthritis (RA) is a chronic systemic autoimmune disease characterized by synovial inflammation, pain, fatigue, progressive joint damage, and functional limitation [1]. The global burden of RA remains substantial, with the Global Burden of Disease Study 2021 estimating 17.6 million prevalent cases in 2020 and projecting an increase to 31.7 million by 2050 [2]. Beyond inflammatory and structural manifestations, RA is also accompanied by a considerable psychological burden. Systematic reviews have reported that depressive symptoms and anxiety are common among people with RA [3,4]. These symptoms warrant clinical attention because they are often associated with greater symptom burden, poorer physical function, and impaired health-related quality of life (HRQoL). Consistently, HRQoL has been shown to be compromised across both physical and mental health domains in people with RA [5]. Therefore, addressing the psychological impact of RA is important for improving HRQoL and patient-centered outcomes in this population.

Exercise and physical activity are recommended as part of comprehensive management for people with inflammatory arthritis, including RA [6]. Previous reviews suggest that exercise may improve physical function, cardiorespiratory fitness, pain, fatigue, and other patient-reported outcomes in RA [7–9]. However, whether exercise interventions improve depressive symptoms, anxiety, and HRQoL in adults with RA remains uncertain. This question is clinically important because exercise participation in RA may be shaped not only by physical capacity and disease activity, but also by pain-related beliefs, confidence in movement, and fear of symptom exacerbation. The fear-avoidance model suggests that pain-related fear may lead to activity avoidance, physical disuse, and disability in chronic musculoskeletal conditions [10]. In RA-specific studies, fear-avoidance beliefs and kinesiophobia-two related forms of pain-related fear-have been associated with greater pain, poorer functional status, depressive symptoms, and reduced quality of life [11,12]. These factors may therefore influence both engagement with exercise and the extent to which exercise interventions improve patient-centered outcomes.

This uncertainty has not been fully addressed by previous reviews, which have mainly focused on physical function, disease activity, pain, fatigue, aerobic capacity, or broader patient-reported outcomes [7–9]. Less attention has been given to depressive symptoms, anxiety, and HRQoL as primary outcomes of exercise interventions in adults with RA. In addition, exercise interventions vary in modality, ranging from conventional exercise such as resistance training and aerobic exercise to mind-body approaches such as Tai Chi and yoga. Previous reviews of Tai Chi and yoga in RA have also shown that the available evidence remains limited and heterogeneous, particularly with respect to psychological outcomes [13,14]. Therefore, a focused synthesis of depressive symptoms, anxiety, and HRQoL, with further consideration of differences between conventional and mind-body exercise, may help clarify the role of exercise in patient-centered RA management.

Accordingly, this systematic review and meta-analysis aimed to evaluate the effects of structured exercise interventions on depressive symptoms, anxiety, and HRQoL in adults with RA. Where sufficient data were available, we further examined whether these effects differed between conventional exercise and mind-body exercise interventions.

## Methods

### Protocol and reporting

This systematic review and meta-analysis was conducted and reported in accordance with the PRISMA 2020 statement [15]. The completed PRISMA 2020 checklist is provided as S1 Checklist. The review protocol was retrospectively registered with INPLASY (INPLASY202610082) after completion of the database search and is provided as S1 Protocol. The protocol described the review question, eligibility criteria, outcomes, intervention categories, risk-of-bias assessment, and planned quantitative syntheses.

Two deviations from the registered protocol should be noted. First, although the registered protocol did not specify language restrictions, only English-language full-text articles were included in the final review because translation resources were not available. This restriction may have introduced language bias and is acknowledged as a limitation. Second, the registered protocol described use of the standard Cochrane risk-of-bias domains, whereas the present review used the Cochrane Risk of Bias 2 tool for randomized trials. This change was made because all included studies were randomized controlled trials and RoB 2 provides a structured, outcome-level assessment for randomized trials. No changes were made to the review question, primary outcomes, intervention categories, comparator definition, or planned quantitative synthesis approach.

### Search strategy

We systematically searched PubMed, Embase, the Cochrane Central Register of Controlled Trials (CENTRAL), Web of Science Core Collection, Scopus, PsycINFO, and an additional EBSCOhost search from database inception to 26 November 2025. The search strategies combined controlled vocabulary and free-text terms related to rheumatoid arthritis, exercise or physical activity, and psychological or quality-of-life outcomes, including depressive symptoms, anxiety, and HRQoL. The complete search strategies for all databases are provided in S1 Table. Reference lists of included studies and relevant reviews were manually screened, and forward citation tracking was performed to identify additional eligible reports. Only articles published in English were eligible for inclusion. Grey literature sources were not systematically searched. Conference-only reports without sufficient outcome data were excluded.

### Eligibility criteria

Studies were eligible if they met the following criteria: (1) randomized controlled trials; (2) adults aged 18 years or older with RA, diagnosed according to established classification criteria, such as ACR or ACR/EULAR criteria, or confirmed by a rheumatologist; (3) evaluation of a structured exercise intervention; (4) inclusion of a non-exercise comparator; and (5) reporting of depressive symptoms, anxiety, or global or summary HRQoL using a validated patient-reported outcome measure.

Structured exercise was defined as planned, repeated physical activity or therapeutic movement intended to improve or maintain physical fitness, physical function, symptoms, or health [16]. Eligible interventions had to include an active movement component and report at least one exercise descriptor, such as exercise type, frequency, session duration, intensity, supervision, progression, or total intervention duration. Conventional exercise included aerobic, resistance, strengthening, flexibility, balance, dance-based, joint-specific, water-based, or mixed exercise programs [6,17]. Mind-body exercise included movement-based practices such as TaiChi, Yoga, Qigong, BaDuanJin, or YiJinJing [18].

Our exclusion criteria were as follows: (1) non-randomized, quasi-randomized, observational, cross-sectional, case-series, protocol-only, or conference-only reports without sufficient outcome data; (2) studies without RA participants, or studies including mixed rheumatic, musculoskeletal, or inflammatory populations without separately extractable RA data; (3) interventions without an active movement component; (4) multimodal interventions in which the independent effect of exercise could not be isolated; (5) comparisons of one exercise intervention only with another exercise intervention without a non-exercise control arm; and (6) studies that did not report eligible psychological or HRQoL outcomes using a validated patient-reported measure. No additional exclusion criterion for severe psychiatric disorders was applied at the review level unless such criteria were specified in the original trial.

### Study selection and data extraction

Two reviewers independently screened titles and abstracts, assessed full texts for eligibility, and extracted data using predefined forms. Disagreements were resolved by discussion or, when necessary, by consultation with a third reviewer. Extracted data included study location, eligibility criteria, sample size, RA diagnostic criteria, intervention type, outcome measures, reporting format, follow-up time points, attrition, and adverse events. The completed data extraction table for the included randomized controlled trials is provided as S2 Table.

When multiple post-intervention assessments were reported, the earliest post-intervention time point was used for the primary analysis to improve comparability across trials. Additional rehabilitation-related information, including supervision, home practice, education or self-management support, adherence, and adverse-event reporting, was extracted when reported to describe the clinical profile of the included trials. Available details are reported descriptively. These variables were used descriptively and were not examined as formal effect modifiers because the number of studies available for each outcome was limited.

### Outcome harmonization

Before synthesis, eligible instruments were classified as assessing depressive symptoms, anxiety, or global/summary HRQoL. If multiple instruments were reported for the same domain within a study, one validated and most domain-specific measure was retained. Global or summary HRQoL measures were prioritized over isolated subscales. Effect directions were harmonized before pooling so that negative SMDs indicated lower depressive symptoms or anxiety, whereas positive SMDs indicated better HRQoL.

### Risk of bias and certainty of evidence

Risk of bias was assessed at the outcome level using the Cochrane Risk of Bias 2 tool (RoB 2) for randomized trials [19]. Risk-of-bias judgments were made for the effect of assignment to the intervention. The overall judgment for each outcome was classified as low risk of bias, some concerns, or high risk of bias according to RoB 2 guidance. Given the nature of exercise interventions, judgments related to deviations from intended interventions and outcome measurement considered the comparator design, the use of self-reported outcomes, and the potential influence of lack of participant, personnel, or outcome-assessor blinding.

The certainty of evidence for each pooled outcome was assessed using the GRADE framework [20]. Evidence certainty was rated by considering risk of bias, inconsistency, indirectness, imprecision, and publication bias, and was summarized separately for depressive symptoms, anxiety, and health-related quality of life.

### Statistical analysis

Random-effects meta-analyses were conducted using Review Manager software (RevMan, version 5.4), with standardized mean differences (SMDs; Hedges’ g) and 95% confidence intervals calculated because the included studies used different instruments to assess the same outcome domains [21]. The pre-to-post changes in the experimental and control groups were pooled to estimate the intervention effects. Change-from-baseline means were calculated as the post-intervention mean minus the baseline mean. The standard deviation (SD) of the change score was calculated using the formula recommended in the Cochrane Handbook for Systematic Reviews of Interventions [21]:


$$\begin{array}{c} SD_{change}=\sqrt{S\overset{\text{2}}{\underset{baseline}{D}}+S\overset{\text{2}}{\underset{post}{D}}-\text{2}Corr\times SD_{baseline}\times SD_{post}} \end{array}$$

where Corr denotes the correlation between baseline and post-intervention measurements.

When a study included multiple eligible exercise arms sharing a single control group, comparable intervention arms were combined according to standard Cochrane methods so that each study contributed only one independent comparison per outcome [21]. Between-study heterogeneity was assessed using the I² statistic [22]. Planned subgroup analyses compared intervention modality, categorized as conventional exercise or mind-body exercise, and were performed only when at least two studies were available in each subgroup. Meta-regression was not conducted because the number of studies per outcome was insufficient and relevant covariates were inconsistently reported.

Sensitivity analyses were conducted using leave-one-out procedures. Small-study effects and potential publication bias were assessed for each outcome. Funnel plots were visually inspected when at least 10 studies were available for a given outcome. Formal tests for funnel plot asymmetry were not performed because the number of studies remained small, with only one outcome reaching the conventional threshold for funnel plot assessment. For outcomes with fewer than 10 studies, publication bias was considered qualitatively and incorporated into the GRADE assessment where relevant.

## Results

### Study selection and study characteristics

**Fig 1:**
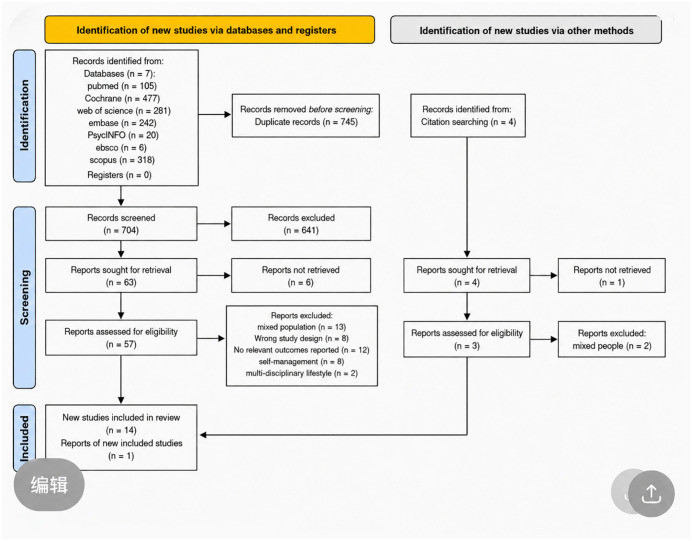
PRISMA flow diagram of study selection.

The study selection process is summarized in Fig 1. Database searching identified 1449 records. After removal of 745 duplicates, 704 titles and abstracts were screened, of which 641 were excluded. Sixty-three reports from database searching were sought for retrieval; 57 were assessed in full text after six reports could not be retrieved. Citation searching identified four additional reports; all four were sought for retrieval, one could not be retrieved, and three were assessed in full text, 60 full-text reports were assessed for eligibility, and 15 randomized controlled trials met the inclusion criteria.

### Study and participant characteristics

The 15 included trials involved 1652 participants and were conducted in Sweden, New Zealand, the United States, India, Denmark, the United Kingdom, China, Canada, Croatia, and the Netherlands. Nine trials evaluated conventional exercise interventions [23–31] and six evaluated mind-body exercise interventions [32–37]. Reported mean or median participant age ranged from 45.7 to 69.1 years, and reported mean or median disease duration ranged from 6.3 to 20 years.

The included trials were clinically heterogeneous in population characteristics, exercise modality, intervention dose, delivery setting, supervision, and comparator type. Several trials were small, with six trials enrolling fewer than 60 participants in total, which should be considered when interpreting the precision and robustness of the pooled estimates. Intervention duration ranged from 6 to 52 weeks, and session frequency ranged from one to seven sessions per week. Conventional exercise programs included aerobic, resistance, strengthening, flexibility, dance-based, joint-specific, water-based, and mixed exercise interventions, whereas mind-body programs included Yoga, TaiChi, and YiJinJing. Comparator conditions included usual care, usual activity, education, lectures, counseling, physiotherapy advice, or low-intensity home advice that did not meet the structured exercise definition used in this review. Detailed study characteristics and available exercise-intervention details, including available exercise-intervention details information where reported, are summarized in Table 1.

**Table 1:** Characteristics of included studies and exercise interventions.

Study ID	Country	Inclusion Criteria/ Severity	N (E/C)	Gender (M/F)E/C	Age (Mean ± SD)	Disease Duration(Mean ± SD)	Intervention (Experimental)	Control Group	Outcomes
Wang; 2008 [32]	USA	ACR functional class I or II	E: 10 C: 10	E: 2/8 C: 3/7	E: 48 ± 10 C: 51 ± 17	E: 14 ± 6 C: 15 ± 11	Tai Chi; FITT: 12 weeks×2 sessions/week; Intensity: Low	Health education lectures	Depression (CES-D) HRQoL (SF-36)
Kucharski; 2019 [23]	Sweden	ACR 1987/ EULAR 2010 criteria; DAS28 < 5.1	E: 36 C: 38	E: 9/27 C: 9/29	E: 69.1 ± 2.6 C: 70.1 ± 3.1	E: >2 years C: NR	Gym-based aerobic & resistance training; FITT: 20 weeks×3 sessions/week; Intensity: Moderate-to-high	Home-based light exercise	Anxiety (HADS) Depression (HADS)
Gautam; 2019 [33]	India	ACR/EULAR 2010 criteria; DAS28-ESR > 2.6	E: 36 C: 36	E: 7/29 C: 9/27	E: 45.7 ± 1.6 C: 42.1 ± 1.7	E: 6.3 ± 0.8 C: 5.6 ± 0.7	Yoga; FITT: 8 weeks×5 sessions/week; Intensity: Low-to-moderate	Usual care	Depression (BDI-II)
Baxter; 2016 [24]	New Zealand	ACR/EULAR 2010 criteria	E: 11 C: 22	E: 1/10 C: 5/17	E: 66.6 ± 10.1 C: 59.4 ± 12.9	E: 8.6 ± 2.3 C: 6.3 ± 4.9	Walking program; FITT: 6 weeks×4 sessions/week; Intensity: Moderate	Nutritional advice	Depression (BDI-II)
Shetty; 2025 [34]	India	ACR/EULAR 2010 criteria; DAS28 > 2.6	E: 50 C: 50	E: 12/38 C: 17/33	E: 49.0 ± 10.0 C: 47.8 ± 9.3	E: 6.7 ± 2.0 C: 6.1 ± 2.6	Integrated Yoga therapy; FITT: 12 weeks×5 sessions/week; Intensity: Low-to-moderate	Regular medication	HRQoL (EQ-5D)
Teuwen; 2024 [25]	Netherlands	Clinical diagnosis by rheumatologist	E: 109 C: 106	E: 12/97 C: 9/97	E: 59.4 ± 12.1 C: 58.1 ± 13.6	E: 18.0 ± 11.9 C: 19.7 ± 14.1	Long-term exercise therapy (multimodal); FITT: 52 weeks×2 sessions/week; Intensity: NR	Usual care	HRQoL (SF-36)
Ward; 2018 [35]	New Zealand	ACR/EULAR 2010 criteria	E: 13 C: 13	E: 0/13 C: 1/12	E: 50 ± 12 C: 59 ± 8	E: 11 ± 10 C: 12 ± 11	Yoga; FITT: 8 weeks×1 session/week; Intensity: Low-to-moderate	Usual care	Anxiety (HADS) Depression (HADS) HRQoL (EQ-5D)
Chang; 2024 [36]	China	ACR 1987 or EULAR 2010 criteria; DAS28-ESR ≤ 3.2	E: 30 C: 30	E: 1/29 C: 2/28	E: 54.8 ± 8.5 C: 51.6 ± 10.6	E: NR C: NR	Yijinjing; FITT: 12 weeks×3 sessions/week; Intensity: Low-to-moderate	Usual care	Anxiety (SAS) Depression (SDS)
Loeppenthin; 2022 [26]	Denmark	DAS28 < 3.2	E: 17 C: 21	E: 4/13 C: 1/20	E: 57.8 ± 9.8 C: 54.8 ± 9.6	E: 10.1 ± 5.9 C: 17.9 ± 12.8	Intermittent aerobic exercise; FITT: 6 weeks×3 sessions/week; Intensity: Moderate-to-high	Usual care	Depression (CES-D)
Bilberg; 2025 [27]	Sweden	ACR 1987 or EULAR 2010 criteria	E: 43 C: 44	E: 6/37 C: 8/36	E: 48.4 ± 10.1 C: 47.9 ± 9.3	E: 11.5 ± 9.5 C: NR	High-intensity interval training (HIIT); FITT: 12 weeks×2 sessions/week; Intensity: High	Physiotherapy counseling	Anxiety (HADS) Depression (HADS)
Pukšić; 2021 [37]	Croatia	ACR/EULAR 2010 criteria; DAS28-CRP < 5.1	E: 30 C: 27	E: 0/30 C: 3/24	E: 52.9 ± 12.2 C: 57.9 ± 9.0	E: 7.4 ± 6.1 C: 8.7 ± 9.2	Yoga in Daily Life program; FITT: 12 weeks×2 sessions/week; Intensity: Low-to-moderate	Weekly lectures	Anxiety (HADS) Depression (HADS) HRQoL (EQ-5D)
Noreau; 1995 [28]	Canada	ACR functional class I or II	E: 19 C: 10	E: 0/19 C: 0/10	E: 49.3 ± 13.0 C: 49.4 ± 11.9	E: 8.1 ± 8.2 C: 11.0 ± 5.1	Aerobic dance; FITT: 12 weeks×2 sessions/week; Intensity: Moderate	Counseling	Anxiety (AIMA/POMA) Depression (AIMA/POMA)
Manning; 2014 [29]	UK	ACR 1987 revised criteria	E: 52 C: 56	E: 8/44 C: 18/38	E: 53 ± 16 C: 57 ± 15	E: 20 ± 18 C: 20 ± 19	Upper extremity exercise & self-management; FITT: 12 weeks×7 sessions/week; Intensity: Moderate-to-high	Education & self-management	HRQoL (RAQoL)
Neuberger; 2007 [30]	USA	ACR 1987 criteria	E: 79 C: 73	E: NR C: NR	E: 55.5 ± 11.2 C: NR	E: 8.0 ± 4.2 C: NR	Home aerobic fitness (video-guided); FITT: 12 weeks×3 sessions/week; Intensity: Moderate-to-high	Usual care	Depression (CES-D)
Feldthusen; 2015 [31]	Sweden	ICD-10 code M05/M06; DAS28 < 3.8	E: 36 C: 34	E: 4/32 C: 4/30	E: 54.2 ± 8.5 C: 52.7 ± 10.9	E: 14.2 ± 11.1 C: 11.6 ± 7.7	Person-centered physical therapy; FITT: 12 weeks×3-5 sessions/week; Intensity: Moderate-to-high	Usual activity	Anxiety (HADS) Depression (HADS) HRQoL (EQ-5D)

Abbreviations: E: Experimental group; C: Control group; NR: Not Reported; M/F: Male/Female; ACR: American College of Rheumatology; EULAR: European Alliance of Associations for Rheumatology; DAS28: Disease Activity Score in 28 joints; FITT: Frequency, Intensity, Time, Type; Outcome measures: HADS: Hospital Anxiety and Depression Scale; CES-D: Center for Epidemiologic Studies Depression Scale; BDI-II: Beck Depression Inventory-II; SF-36: Short Form 36 Health Survey; EQ-5D: EuroQol-5D; SAS/SDS: Self-rating Anxiety/Depression Scale.

Depressive symptoms were reported in 11 trials, anxiety in seven trials, and global or summary HRQoL in seven trials. Psychological and HRQoL outcomes were measured using validated patient-reported instruments. Depressive symptoms were assessed using HADS-D, BDI-II, CES-D, and SDS; anxiety was assessed mainly using HADS-A or SAS or AIMA/POMA; and HRQoL was assessed using SF-36, EQ-5D-3L, RAQoL, and VAS-Global. MCID thresholds or proportions of participants achieving clinically important improvement were not consistently reported across trials; therefore, pooled SMDs were interpreted as standardized effects rather than direct estimates of clinically important improvement.

### Risk of bias

**Fig 2:**
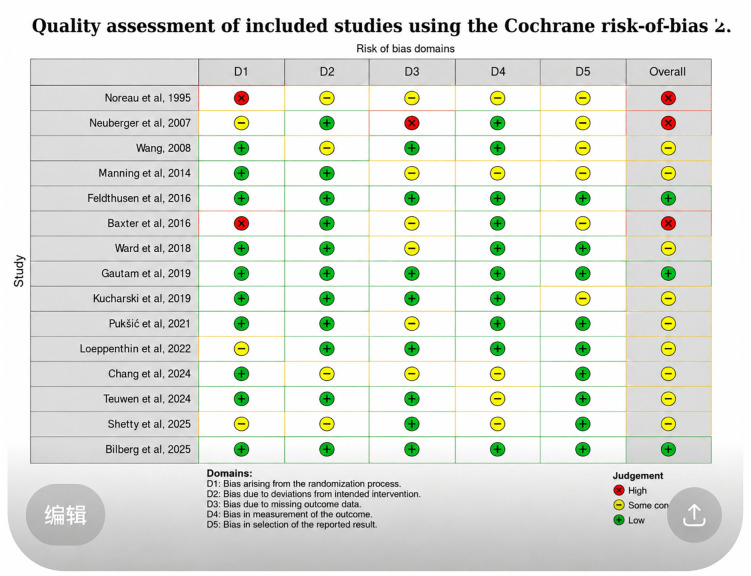
Risk of bias assessment.

Risk-of-bias assessments using the Cochrane Risk of Bias 2 tool are presented in Fig 2. Overall, the most frequent concerns were related to deviations from intended interventions and measurement of outcomes. Because exercise interventions are behavioral in nature, blinding of participants and intervention personnel was generally infeasible. In addition, depressive symptoms, anxiety, and HRQoL were mainly assessed using self-reported questionnaires, which may have increased the risk of expectancy or response bias.

Concerns related to the randomization process, missing outcome data, and selection of the reported result varied across trials. In several studies, reporting detail was insufficient to fully judge allocation procedures or prespecification of outcomes. Missing outcome data were not consistently reported across all trials, although they were generally not the main source of concern.

### Effects on depressive symptoms

Eleven trials involving 685 participants reported depressive symptom outcomes. In a random-effects meta-analysis, exercise was associated with lower depressive symptom scores than non-exercise control conditions, expressed as Hedges’g standardized mean difference (SMD = −0.49; 95% CI, −0.70 to −0.28; P < 0.001). Statistical heterogeneity was moderate (I² = 42%). Depression outcomes were measured using different instruments, and minimally important change thresholds were not consistently reported in the primary trials.

**Fig 3:**
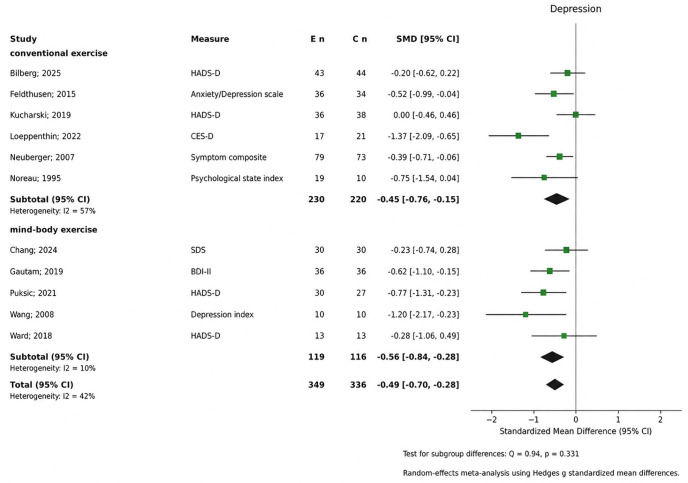
Forest plot of the effect of exercise-based rehabilitation on depressive symptoms.

In subgroup analyses by exercise modality, both conventional exercise and mind-body exercise were associated with lower depressive symptom scores, with no statistically significant subgroup difference (Chi² = 0.24; P = 0.62) (Fig 3).

### Effects on anxiety

Seven trials involving 403 participants reported anxiety outcomes. In a random-effects meta-analysis, exercise was associated with lower anxiety scores than control conditions, expressed as Hedges’g standardized mean difference (SMD = −0.45; 95% CI, −0.65 to −0.25; P < 0.001). Statistical heterogeneity was low (I² = 0%). Anxiety was most commonly assessed using the HADS-A, with other instruments including the SAS, an anxiety scale, and a psychological state index.

**Fig 4:**
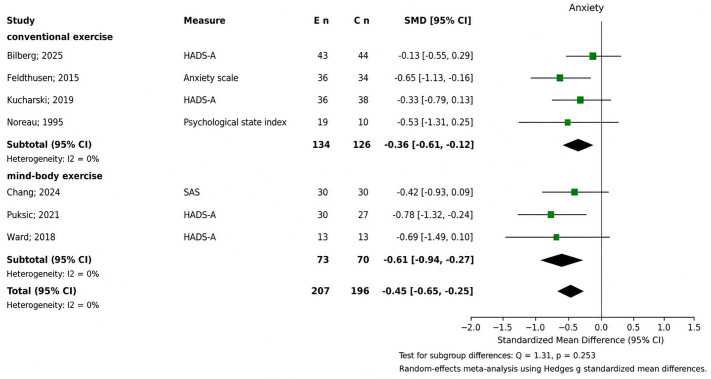
Forest plot of the effect on anxiety.

No statistically significant subgroup difference was observed between conventional exercise and mind-body exercise for anxiety (Chi² = 1.29; P = 0.26) (Fig 4).

### Effects on health-related quality of life

Seven trials involving 564 participants reported quality-of-life or health-related quality-of-life (QoL/HRQoL) outcomes and were included in the quantitative synthesis. QoL/HRQoL was assessed using validated disease-specific and generic instruments, including the RAQoL, EuroQol/EQ-5D-related measures, and the SF-36. In a random-effects meta-analysis, exercise was associated with significantly improved QoL/HRQoL compared with control conditions, expressed as Hedges’g standardized mean difference (SMD = 0.45; 95% CI, 0.18 to 0.71; P = 0.001). Statistical heterogeneity was moderate (I² = 50%).

**Fig 5:**
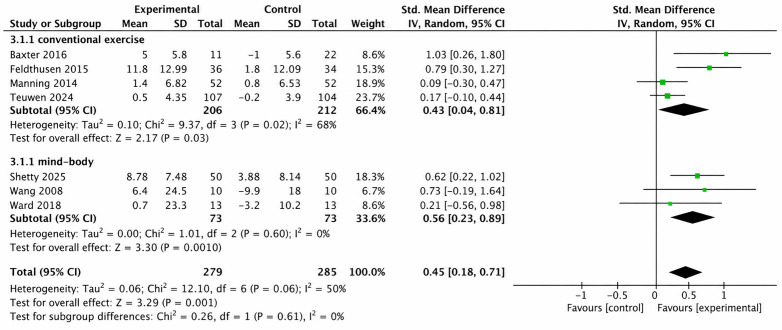
Forest plot of the effect on health-related quality of life.

Subgroup analyses by exercise modality showed significant improvements in QoL/HRQoL for both conventional exercise and mind-body exercise. The pooled effect estimate for conventional exercise was SMD = 0.43 (95% CI, 0.04 to 0.81; I² = 68%), whereas the pooled effect estimate for mind-body exercise was SMD = 0.56 (95% CI, 0.23 to 0.89; I² = 0%). No statistically significant subgroup difference was observed between conventional exercise and mind-body exercise for QoL/HRQoL (Chi² = 0.26; P = 0.61) (Fig 5).

### Small-study effects, adverse events, and certainty of evidence

**Fig 6:**
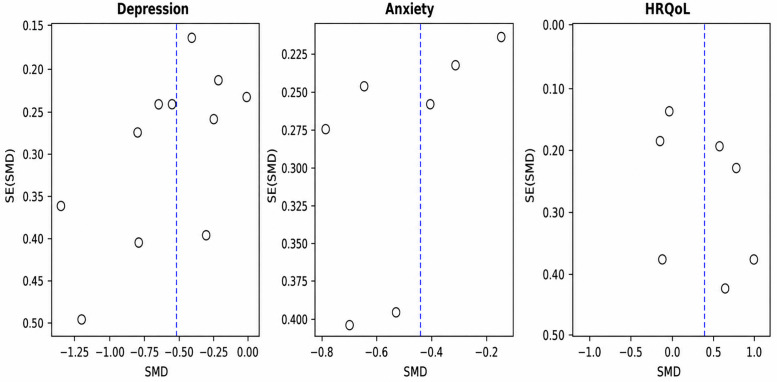
Funnel plot for publication bias.

The number of studies contributing to each meta-analysis was modest. Funnel plots were visually inspected to explore potential small-study effects (Fig 6). For depressive symptoms, the funnel plot did not suggest marked asymmetry. However, small-study effects and publication bias could not be excluded because only 11 trials contributed to this outcome, limiting the reliability of visual assessment and the power of formal asymmetry testing. Funnel plots for anxiety and HRQoL were presented descriptively only, as fewer than 10 studies contributed to these outcomes. Formal asymmetry testing was therefore not performed.

Adverse-event information was available for 11 trials, although the level of reporting detail varied. No serious exercise-related adverse events were clearly documented. Reported intervention-related events were generally minor and included transient musculoskeletal pain, positional vertigo during yoga, and irregular heart rate during high-intensity interval training; this episode was managed by modifying exercise intensity rather than discontinuing participation.

Using GRADE, the certainty of evidence was rated as moderate for depressive symptoms and low for anxiety and HRQoL (Table 2). The main reasons for downgrading were risk of bias related to deviations from intended interventions and self-reported outcome measurement, imprecision due to small sample sizes and few contributing trials, and limited ability to assess publication bias.

**Table 2:** GRADE Summary of Evidence Quality and Effect Estimates for Depression, Anxiety, and Quality of Life Outcomes.

Outcome	No. of studies	No. of participants	Study Design	Risk of Bias	Inconsistency	Indirectness	Imprecision	Publication bias	Effect estimate	Heterogeneity	Certainty	Importance
Depression	11	685	RCTs	Serious	Not Serious	Not Serious	Not Serious	Not Serious	SMD = −0.49; 95% CI −0.70 to −0.28; P < 0.001	I² = 42%	Moderate	Critical
Anxiety	7	403	RCTs	Serious	Not Serious	Not Serious	Serious	Not Serious	SMD = −0.45; 95% CI −0.65 to −0.25; P < 0.001	I² = 0%	Low	Important
Quality of Life (QoL)	7	564	RCTs	Serious	Not Serious	Not Serious	Serious	Not Serious	SMD = 0.45; 95% CI 0.18 to 0.71; P = 0.001	I² = 50%	Low	Critical

Abbreviations: CI, confidence interval; GRADE, Grading of Recommendations Assessment, Development and Evaluation; HRQoL, health-related quality of life; RCT, randomized controlled trial; SMD, standardized mean difference.

## Discussion

This systematic review and meta-analysis found that structured exercise interventions were associated with reductions in depressive symptoms and anxiety and with improvements in health-related quality of life (HRQoL) among adults with rheumatoid arthritis (RA). The certainty of evidence was moderate for depressive symptoms and low for anxiety and HRQoL. These findings suggest that exercise may provide psychological and quality-of-life benefits as an adjunct to standard RA care. However, current evidence is insufficient to identify the most effective exercise modality, dose, supervision strategy, or delivery format.

This review extends previous syntheses of exercise interventions in RA by focusing specifically on depressive symptoms, anxiety, and HRQoL. Prior reviews have primarily emphasized physical function, pain, fatigue, aerobic capacity, disease activity, or broader patient-reported outcomes [38,39]. This focus is clinically relevant because psychological symptoms in RA frequently coexist with pain, fatigue, reduced participation, and functional limitation [4,7,8], and may persist despite otherwise appropriate pharmacologic disease control [10,12]. Exercise-based rehabilitation may therefore contribute to multidimensional RA management by supporting not only physical capacity, but also confidence, participation, symptom coping, and perceived health.

Although the pooled effects were generally favorable, their clinical interpretation depends on the measurement instruments used and the availability of clinically meaningful thresholds. Standardized mean differences were appropriate because the included trials used different instruments to assess similar constructs, but they are less intuitive for clinical decision-making. Although back-translation and minimal clinically important difference or minimally important difference thresholds can aid clinical interpretation, such interpretation was limited in this review because thresholds vary by instrument, population, baseline severity, and anchor method [40,41]. Moreover, most trials did not report the proportion of participants achieving clinically meaningful improvement. Therefore, although the results support the potential benefits of exercise interventions, the extent to which these changes translate into patient-perceived clinical improvement requires further investigation.

Interpretation of the HRQoL findings is influenced by the limited number of contributing studies and heterogeneity in measurement instruments. Only seven studies provided global or summary HRQoL data suitable for quantitative pooling, and these studies used heterogeneous measures, including generic health-status indices, SF-36 summary scores, and global quality-of-life ratings. These instruments capture related but not identical constructs. Thus, the HRQoL findings are best understood as supportive evidence that structured exercise may improve perceived health status, rather than definitive evidence that all quality-of-life domains improve consistently.

The included trials were clinically heterogeneous with respect to exercise type, frequency, intensity, duration, progression, supervision, delivery setting, home-practice requirements, and comparator conditions. Accordingly, the pooled estimates should be understood as average effects of structured exercise programs rather than evidence for a single optimal prescription. The absence of statistically significant subgroup differences between conventional and mind-body exercise should not be interpreted as evidence of equivalence. Both categories included diverse interventions, and subgroup analyses were limited by small study numbers and within-category heterogeneity. These findings support flexibility in exercise selection but do not establish that all exercise formats are interchangeable.

Several plausible mechanisms may explain the observed benefits. Exercise may improve depressive symptoms and anxiety by enhancing physical function, sleep, symptom coping, self-efficacy, behavioral activation, and confidence in movement. These pathways are particularly relevant in RA, where pain-related fear, low confidence, and uncertainty about symptom exacerbation may reduce activity participation and reinforce cycles of inactivity and low mood. Mind-body exercise may be acceptable for some patients because it combines low-impact movement with breathing, attentional focus, and relaxation [18,42]. However, mechanistic variables were rarely measured directly in the included trials. These explanations should therefore be regarded as hypotheses rather than confirmed causal pathways.

This review has several limitations. The included trials were generally small and varied substantially in intervention characteristics, comparator conditions, and outcome instruments. Most psychological and HRQoL outcomes were self-reported, and participant blinding was usually infeasible, increasing susceptibility to bias related to deviations from intended interventions and outcome measurement. The number of studies contributing to each outcome was limited, reducing the precision of subgroup analyses and limiting the ability to assess publication bias, particularly for anxiety and HRQoL. Most analyses used immediate post-intervention outcomes because long-term follow-up data were limited; therefore, the durability of benefit remains uncertain. Incomplete reporting of participant flow, adverse events, arm-level outcome data, and predefined or protocol-specified outcomes in the primary trials also limited verification and quantitative synthesis for some outcomes.

Future RA exercise trials should prespecify psychological and HRQoL outcomes, enroll samples large enough to detect clinically meaningful differences, report complete arm-level data at each time point, and include longer follow-up. Trials should also report the proportion of participants achieving clinically meaningful improvement when validated thresholds are available. More consistent use of intervention-reporting frameworks such as TIDieR and CERT would improve reproducibility by clarifying exercise progression, supervision, adherence support, home-practice content, and safety monitoring [43,44]. Future studies should also measure potential mediators, such as self-efficacy, fear of movement, sleep, fatigue, and activity participation, to clarify how exercise may influence psychological and quality-of-life outcomes in RA.

Taken together, these findings support structured exercise as a reasonable adjunctive strategy for improving depressive symptoms, anxiety, and perceived health status in adults with RA. The evidence is promising but not definitive. Exercise should be integrated into comprehensive RA care where appropriate, rather than viewed as a substitute for pharmacologic disease control or formal mental-health care when these are indicated. Future trials should move beyond asking whether exercise helps and clarify which approaches work best, for whom, through which mechanisms, and with what durability.

## Conclusions

In adults with rheumatoid arthritis, structured exercise interventions were associated with favorable effects on depressive symptoms, anxiety, and health-related quality of life compared with non-exercise control conditions. These findings support structured exercise as an adjunctive component of comprehensive RA care, including for patients with persistent psychological distress, reduced activity confidence, limited participation, or impaired perceived health. Exercise should not be viewed as a substitute for pharmacologic disease control or formal mental-health care when these are indicated.

Current evidence does not establish the superiority of any specific exercise modality or delivery format. Exercise selection should therefore be individualized according to symptoms, functional limitations, patient preferences, access to supervision, home-practice feasibility, and safety considerations. The certainty of evidence was moderate for depressive symptoms and low for anxiety and health-related quality of life, reflecting small study numbers, self-reported outcomes, limited blinding, and heterogeneity across interventions.

Larger and better-reported trials with longer follow-up are needed to clarify the magnitude, durability, and clinical targeting of psychological benefit. Future studies should report complete intervention, adherence, safety, comparator, and clinically interpretable outcome data, including the proportion of participants achieving minimally important improvement.

## Supporting information

S1 Checklist PRISMA 2020 checklist. (DOCX)

S1 Table Search strategy. (DOCX)

S2 Table Data extraction table. (XLSX)

S1 Protocol Registered INPLASY protocol. (PDF)
