# Supplementary material for: Effectiveness of exercise-based rehabilitation for depressive symptoms, anxiety, and health-related quality of life in adults with rheumatoid arthritis: A systematic review and meta-analysis of randomized controlled trials
Source: PLoS One. 2026 Jul 15;21(7):e0352173. doi: 10.1371/journal.pone.0352173 (PMC13372145; doi:10.1371/journal.pone.0352173)
Supplement: S1 Checklist — (DOCX) [file pone.0352173.s001.docx]

| **Section and Topic** | **Item #** | **Checklist item** | **Location where item is reported** |
| --- | --- | --- | --- |
| **TITLE** | | |  |
| Title | 1 | Identify the report as a systematic review. | Title, page 1. |
| **ABSTRACT** | | |  |
| Abstract | 2 | See the PRISMA 2020 for Abstracts checklist. | Abstract, pages 4-7. |
| **INTRODUCTION** | | |  |
| Rationale | 3 | Describe the rationale for the review in the context of existing knowledge. | Introduction-Rationale, pages 8-12. |
| Objectives | 4 | Provide an explicit statement of the objective(s) or question(s) the review addresses. | Introduction-Objectives, page 12. |
| **METHODS** | | |  |
| Eligibility criteria | 5 | Specify the inclusion and exclusion criteria for the review and how studies were grouped for the syntheses. | Methods-Eligibility criteria, pages 16-19; Statistical analysis, pages 23-25. |
| Information sources | 6 | Specify all databases, registers, websites, organisations, reference lists and other sources searched or consulted to identify studies. Specify the date when each source was last searched or consulted. | Methods-Search strategy, pages 15-16; S1 Table. |
| Search strategy | 7 | Present the full search strategies for all databases, registers and websites, including any filters and limits used. | Methods-Search strategy, pages 15-16; S1 Table. |
| Selection process | 8 | Specify the methods used to decide whether a study met the inclusion criteria of the review, including how many reviewers screened each record and each report retrieved, whether they worked independently, and if applicable, details of automation tools used in the process. | Methods-Study selection and data extraction, pages 19-21. |
| Data collection process | 9 | Specify the methods used to collect data from reports, including how many reviewers collected data from each report, whether they worked independently, any processes for obtaining or confirming data from study investigators, and if applicable, details of automation tools used in the process. | Methods-Study selection and data extraction, pages 19-21. |
| Data items | 10a | List and define all outcomes for which data were sought. Specify whether all results that were compatible with each outcome domain in each study were sought (e.g. for all measures, time points, analyses), and if not, the methods used to decide which results to collect. | Methods-Eligibility criteria, pages 16-19; Outcome harmonization, pages 21-22. |
|  | 10b | List and define all other variables for which data were sought (e.g. participant and intervention characteristics, funding sources). Describe any assumptions made about any missing or unclear information. | Methods-Study selection and data extraction, pages 19-21; Table 1, pages 31-33; S2 Table. |
| Study risk of bias assessment | 11 | Specify the methods used to assess risk of bias in the included studies, including details of the tool(s) used, how many reviewers assessed each study and whether they worked independently, and if applicable, details of automation tools used in the process. | Methods-Risk of bias and certainty of evidence, pages 22-23. |
| Effect measures | 12 | Specify for each outcome the effect measure(s) (e.g. risk ratio, mean difference) used in the synthesis or presentation of results. | Methods-Statistical analysis, pages 23-25. |
| Synthesis methods | 13a | Describe the processes used to decide which studies were eligible for each synthesis (e.g. tabulating the study intervention characteristics and comparing against the planned groups for each synthesis (item #5)). | Methods-Eligibility criteria, Outcome harmonization, and Statistical analysis, pages 16-25. |
|  | 13b | Describe any methods required to prepare the data for presentation or synthesis, such as handling of missing summary statistics, or data conversions. | Methods-Outcome harmonization and Statistical analysis, pages 21-25. |
|  | 13c | Describe any methods used to tabulate or visually display results of individual studies and syntheses. | Methods-Statistical analysis, pages 23-25; Results-Table 1, pages 31-33 and Figs 3-5, pages 37-39. |
|  | 13d | Describe any methods used to synthesize results and provide a rationale for the choice(s). If meta-analysis was performed, describe the model(s), method(s) to identify the presence and extent of statistical heterogeneity, and software package(s) used. | Methods-Statistical analysis, pages 23-25. |
|  | 13e | Describe any methods used to explore possible causes of heterogeneity among study results (e.g. subgroup analysis, meta-regression). | Methods-Statistical analysis, pages 24-25; Results-outcome subgroup analyses, pages 37-39. |
|  | 13f | Describe any sensitivity analyses conducted to assess robustness of the synthesized results. | Methods-Statistical analysis, page 25. |
| Reporting bias assessment | 14 | Describe any methods used to assess risk of bias due to missing results in a synthesis (arising from reporting biases). | Methods-Statistical analysis, page 25; Results-Small-study effects, adverse events, and certainty of evidence, pages 40-43. |
| Certainty assessment | 15 | Describe any methods used to assess certainty (or confidence) in the body of evidence for an outcome. | Methods-Risk of bias and certainty of evidence, pages 22-23. |
| **RESULTS** | | |  |
| Study selection | 16a | Describe the results of the search and selection process, from the number of records identified in the search to the number of studies included in the review, ideally using a flow diagram. | Results-Study selection and study characteristics, pages 26-27; Fig 1. |
|  | 16b | Cite studies that might appear to meet the inclusion criteria, but which were excluded, and explain why they were excluded. | Results-Study selection and study characteristics, pages 26-27; Fig 1. |
| Study characteristics | 17 | Cite each included study and present its characteristics. | Results-Study and participant characteristics, pages 27-33; Table 1; Refs 23-37. |
| Risk of bias in studies | 18 | Present assessments of risk of bias for each included study. | Results-Risk of bias, pages 35-36; Fig 2. |
| Results of individual studies | 19 | For all outcomes, present, for each study: (a) summary statistics for each group (where appropriate) and (b) an effect estimate and its precision (e.g. confidence/credible interval), ideally using structured tables or plots. | Results-Outcome syntheses, pages 36-40; Figs 3-5; S2 Table. |
| Results of syntheses | 20a | For each synthesis, briefly summarise the characteristics and risk of bias among contributing studies. | Results-Study characteristics/Risk of bias, pages 27-36; outcome syntheses, pages 36-40. |
|  | 20b | Present results of all statistical syntheses conducted. If meta-analysis was done, present for each the summary estimate and its precision (e.g. confidence/credible interval) and measures of statistical heterogeneity. If comparing groups, describe the direction of the effect. | Results-Depressive symptoms, anxiety, and HRQoL, pages 36-40; Figs 3-5. |
|  | 20c | Present results of all investigations of possible causes of heterogeneity among study results. | Results-outcome subgroup analyses, pages 37-39; Figs 3-5. |
|  | 20d | Present results of all sensitivity analyses conducted to assess the robustness of the synthesized results. | Not separately reported in Results; Methods-Statistical analysis describes leave-one-out sensitivity analyses, page 25. |
| Reporting biases | 21 | Present assessments of risk of bias due to missing results (arising from reporting biases) for each synthesis assessed. | Results-Small-study effects, adverse events, and certainty of evidence, pages 40-43; Fig 6. |
| Certainty of evidence | 22 | Present assessments of certainty (or confidence) in the body of evidence for each outcome assessed. | Results-Certainty of evidence, pages 42-43; Table 2. |
| **DISCUSSION** | | |  |
| Discussion | 23a | Provide a general interpretation of the results in the context of other evidence. | Discussion, pages 43-53. |
|  | 23b | Discuss any limitations of the evidence included in the review. | Discussion-limitations of included evidence/review, pages 50-52. |
|  | 23c | Discuss any limitations of the review processes used. | Methods-Protocol and reporting/Search strategy, pages 13-16; Discussion-limitations, pages 50-52. |
|  | 23d | Discuss implications of the results for practice, policy, and future research. | Discussion and Conclusions, pages 52-55. |
| **OTHER INFORMATION** | | |  |
| Registration and protocol | 24a | Provide registration information for the review, including register name and registration number, or state that the review was not registered. | Methods-Protocol and reporting, pages 13-14. |
|  | 24b | Indicate where the review protocol can be accessed, or state that a protocol was not prepared. | Methods-Protocol and reporting, pages 13-14; S1 Protocol. |
|  | 24c | Describe and explain any amendments to information provided at registration or in the protocol. | Methods-Protocol and reporting, pages 13-14. |
| Support | 25 | Describe sources of financial or non-financial support for the review, and the role of the funders or sponsors in the review. | Funding, page 56. |
| Competing interests | 26 | Declare any competing interests of review authors. | Competing interests, page 56. |
| Availability of data, code and other materials | 27 | Report which of the following are publicly available and where they can be found: template data collection forms; data extracted from included studies; data used for all analyses; analytic code; any other materials used in the review. | Data Availability Statement, page 55; Supporting information captions, page 65; Supporting Information files including S2 Table. |

*From:*  Page MJ, McKenzie JE, Bossuyt PM, Boutron I, Hoffmann TC, Mulrow CD, et al. The PRISMA 2020 statement: an updated guideline for reporting systematic reviews. BMJ 2021;372:n71. doi: 10.1136/bmj.n71. This work is licensed under CC BY 4.0. To view a copy of this license, visit https://creativecommons.org/licenses/by/4.0/
