# Supplementary material for: Effectiveness of exercise-based rehabilitation for depressive symptoms, anxiety, and health-related quality of life in adults with rheumatoid arthritis: A systematic review and meta-analysis of randomized controlled trials
Source: PLoS One. 2026 Jul 15;21(7):e0352173. doi: 10.1371/journal.pone.0352173 (PMC13372145; doi:10.1371/journal.pone.0352173)
Supplement: S1 Table — (DOCX) [file pone.0352173.s002.docx]

# Supplement Contents (Appendix)

**Table 1. Full Search Strategies**

**PubMed**

| #1 | "Rheumatoid Arthritis"[Title/Abstract] OR RA[Title/Abstract] |
| --- | --- |
| #2 | Exercis*[Title/Abstract] OR "Physical Activity"[Title/Abstract] OR sport*[Title/Abstract] OR train*[Title/Abstract] OR fitness[Title/Abstract] OR PA[Title/Abstract] OR aerobic*[Title/Abstract] OR walk*[Title/Abstract] OR jogging[Title/Abstract] OR cycl*[Title/Abstract] OR "high intensity interval"[Title/Abstract] OR HIIT[Title/Abstract] OR swim*[Title/Abstract] OR "Resistance Training"[Title/Abstract] OR strength[Title/Abstract] OR body-mind[Title/Abstract] OR pilates[Title/Abstract] OR Yoga[Title/Abstract] OR "Tai Ji"[Title/Abstract] OR Qigong[Title/Abstract] OR Baduanjin[Title/Abstract] OR Yijinjing[Title/Abstract] OR Wuqinxi[Title/Abstract] OR "Traditional Chinese exercise"[Title/Abstract] |
| #3 | depress*[Title/Abstract] OR anxi*[Title/Abstract] OR burden*[Title/Abstract] OR stress*[Title/Abstract] OR "quality of life"[Title/Abstract] |

**Cochrane**

| #1 | (Rheumatoid Arthritis):ti,ab,kw OR (RA):ti,ab,kw |
| --- | --- |
| #2 | (Exercis*):ti,ab,kw OR ("Physical Activity"):ti,ab,kw OR (sport*):ti,ab,kw OR (train*):ti,ab,kw OR (fitness):ti,ab,kw OR (PA):ti,ab,kw OR (aerobic*):ti,ab,kw OR (walk*):ti,ab,kw OR (jogging):ti,ab,kw OR (cycl*):ti,ab,kw OR ("high intensity interval"):ti,ab,kw OR (HIIT):ti,ab,kw OR (swim*):ti,ab,kw OR ("Resistance Training"):ti,ab,kw OR (strength):ti,ab,kw OR ("body-mind"):ti,ab,kw OR (pilates):ti,ab,kw OR (Yoga):ti,ab,kw OR ("Tai Ji"):ti,ab,kw OR (Qigong):ti,ab,kw OR (Baduanjin):ti,ab,kw OR (Yijinjing):ti,ab,kw OR (Wuqinxi):ti,ab,kw OR ("Traditional Chinese exercise"):ti,ab,kw |
| #3 | (depress*):ti,ab,kw OR (anxi*):ti,ab,kw OR (burden*):ti,ab,kw OR (stress*):ti,ab,kw OR ("quality of life"):ti,ab,kw |

**Web of Science**

| #1 | TS=("Rheumatoid Arthritis") OR TS=(RA) |
| --- | --- |
| #2 | TS=(Exercis*) OR TS=("Physical Activity") OR TS=(sport*) OR TS=(train*) OR TS=(fitness) OR TS=(PA) OR TS=(aerobic*) OR TS=(walk*) OR TS=(jogging) OR TS=(cycl*) OR TS=("high intensity interval") OR TS=(HIIT) OR TS=(swim*) OR TS=("Resistance Training") OR TS=(strength) OR TS=("body-mind") OR TS=(pilates) OR TS=(Yoga) OR TS=("Tai Ji") OR TS=(Qigong) OR TS=(Baduanjin) OR TS=(Yijinjing) OR TS=(Wuqinxi) OR TS=("Traditional Chinese exercise") |
| #3 | TS=(depress*) OR TS=(anxi*) OR TS=(burden*) OR TS=(stress*) OR TS=("quality of life") |

**Embase**

| #1 | "Rheumatoid Arthritis":ti,ab OR "RA":ti,ab |
| --- | --- |
| #2 | "Exercis*":ti,ab OR "Physical Activity":ti,ab OR "sport*":ti,ab OR "train*":ti,ab OR "fitness":ti,ab OR "PA":ti,ab OR "aerobic*":ti,ab OR "walk*":ti,ab OR "jogging":ti,ab OR "cycl*":ti,ab OR "high intensity interval":ti,ab OR "HIIT":ti,ab OR "swim*":ti,ab OR "Resistance Training":ti,ab OR "strength":ti,ab OR "body-mind":ti,ab OR "pilates":ti,ab OR "Yoga":ti,ab OR "Tai Ji":ti,ab OR "Qigong":ti,ab OR "Baduanjin":ti,ab OR "Yijinjing":ti,ab OR "Wuqinxi":ti,ab OR "Traditional Chinese exercise":ti,ab |
| #3 | "depress*":ti,ab OR "anxi*":ti,ab OR "burden*":ti,ab OR "stress*":ti,ab OR "quality of life":ti,ab |

**PsycINFO**

| #1 | "Rheumatoid Arthritis" OR RA |
| --- | --- |
| #2 | Exercis* OR "Physical Activity" OR sport* OR train* OR fitness OR PA OR aerobic* OR walk* OR jogging OR cycl* OR "high intensity interval" OR HIIT OR swim* OR "Resistance Training" OR strength OR "body-mind" OR pilates OR Yoga OR "Tai Ji" OR Qigong OR Baduanjin OR Yijinjing OR Wuqinxi OR "Traditional Chinese exercise" |
| #3 | depress* OR anxi* OR burden* OR stress* OR "quality of life" |

**Scopus**

| #1 | "Rheumatoid Arthritis" OR "RA" |
| --- | --- |
| #2 | "Exercis*" OR "Physical Activity" OR "sport*" OR "train*" OR "fitness" OR "PA" OR "aerobic*" OR "walk*" OR "jogging" OR "cycl*" OR "high intensity interval" OR "HIIT" OR "swim*" OR "Resistance Training" OR "strength" OR "body-mind" OR "pilates" OR "Yoga" OR "Tai Ji" OR "Qigong" OR "Baduanjin" OR "Yijinjing" OR "Wuqinxi" OR "Traditional Chinese exercise" |
| #3 | "depress*" OR "anxi*" OR "burden*" OR "stress*" OR "quality of life" |

**Ebsco**

| #1 | AB "Rheumatoid Arthritis" OR AB RA |
| --- | --- |
| #2 | AB Exercis* OR AB "Physical Activity" OR AB sport* OR AB train* OR AB fitness OR AB PA OR AB aerobic* OR AB walk* OR AB jogging OR AB cycl* OR AB "high intensity interval" OR AB HIIT OR AB swim* OR AB "Resistance Training" OR AB strength OR AB body-mind OR AB pilates OR AB Yoga OR AB "Tai Ji" OR AB Qigong OR AB Baduanjin OR AB Yijinjing OR AB Wuqinxi OR AB "Traditional Chinese exercise" |
| #3 | AB depress* OR AB anxi* OR AB burden* OR AB stress* OR AB "quality of life" |
