# Supplementary material for: Effectiveness of exercise-based rehabilitation for depressive symptoms, anxiety, and health-related quality of life in adults with rheumatoid arthritis: A systematic review and meta-analysis of randomized controlled trials
Source: PLoS One. 2026 Jul 15;21(7):e0352173. doi: 10.1371/journal.pone.0352173 (PMC13372145; doi:10.1371/journal.pone.0352173)
Supplement: S1 Protocol — (PDF) [file pone.0352173.s004.pdf]

International Platform of Registered Systematic Review and Meta-analysis Protocols

Xiao et al. INPLASY protocol 202610082. doi:10.37766/inplasy2026.1.0082 Downloaded from https://inplasy.com/inplasy-2026-1-0082/

INPLASY

INPLASY202610082

doi: 10.37766/inplasy2026.1.0082

Received: 24 January 2026

Published: 24 January 2026

Corresponding author:
xiao yan

24008016003@cupes.edu.cn

Author Affiliation:
Capital University of Physical Education and Sports.

Exercise Interventions for Depression, Anxiety, and Health-Related Quality of Life in Patients With Rheumatoid Arthritis: A Systematic Review and Meta-Analysis of Randomized Clinical Trials

Xiao, Y; Zhong, JH; Yang, XS; Zhang, KB.

ADMINISTRATIVE INFORMATION

Support - No.

Review Stage at time of this submission - Completed but not published.

Conflicts of interest - None declared.

INPLASY registration number: INPLASY202610082

Amendments - This protocol was registered with the International Platform of Registered Systematic Review and Meta-Analysis Protocols (INPLASY) on 24 January 2026 and was last updated on 24 January 2026.

INTRODUCTION

Review question / Objective To evaluate the associations of exercise interventions with depressive symptoms, anxiety, and health-related quality of life in adults with rheumatoid arthritis, and to examine whether associations differ between mind-body and conventional exercise.

Participants/population: Adults (≥18 years) with a diagnosis of rheumatoid arthritis (RA). Intervention(s), exposure(s): Structured exercise interventions (including conventional exercise and mind-body exercise). Comparator(s)/control: Non-exercise controls (e.g., usual care, waiting list, health education, or no intervention). Types of study to be included: Randomized controlled trials (RCTs). Main outcome(s): Depressive symptoms, anxiety, and health-related quality of life.

Rationale Depression and anxiety are common comorbidities in rheumatoid arthritis (RA) that amplify pain and fatigue, yet they are often undertreated in routine care. While exercise is widely recommended for improving physical function in RA, evidence regarding its specific effects on psychological outcomes remains scattered. Furthermore, it is unclear whether different exercise modalities (e.g., mind-body exercises like Tai Chi/Yoga vs. conventional exercises like aerobics/resistance training) offer differential benefits for mental health. This systematic review and meta-analysis is needed to synthesize current evidence from randomized clinical trials to inform more precise, individualized exercise prescriptions for psychological well-being in RA patients.

Condition being studied Rheumatoid arthritis (RA) and its associated psychological comorbidities (specifically depression and anxiety) and health-related quality of life.

METHODS

Search strategy The following electronic databases will be searched from their inception

through November 2025: PubMed, Embase, Cochrane Central Register of Controlled Trials (CENTRAL), Web of Science, Scopus, PsycINFO, and EBSCOhost. The search strategy combines controlled vocabulary (e.g., MeSH terms) and free-text keywords related to three main concepts:

Rheumatoid Arthritis (e.g., "Rheumatoid Arthritis", “RA");

Exercise (e.g., "Exercise", "Physical Activity", "Aerobic", "Resistance Training", "Yoga", "Tai Chi", “Qigong");

Psychological outcomes (e.g., "Depression", "Anxiety", "Quality of Life"). Reference lists of included studies and relevant reviews will be hand-searched, and forward citation tracking will be performed. No restrictions will be imposed regarding publication language or date.

Participant or population This review will include adults (aged 18 years or older) with a clinically diagnosed rheumatoid arthritis (RA). Diagnosis should be based on established classification criteria, such as the 2010 American College of Rheumatology (ACR)/European Alliance of Associations for Rheumatology (EULAR) criteria or earlier standards (e.g., 1987 ACR criteria). Studies involving mixed populations (e.g., mixed arthritis types including osteoarthritis or ankylosing spondylitis) will be excluded unless data for RA participants are reported separately.

Intervention The review will include structured exercise interventions defined as physical training that exceeds routine daily activity. Interventions can be supervised or home-based. Eligible interventions include:

Conventional exercise: Aerobic training, resistance training, combined programs, water-based exercise, etc.

Mind-body exercise: Tai Chi, Yoga, Qigong, Yi Jin Jing, Ba Duan Jin, etc. Multimodal programs combining exercise with other active treatments (e.g., psychotherapy, medication adjustments) will be excluded unless the independent effect of exercise can be isolated (i.e., the control group received the same non-exercise co-intervention).

Comparator The review will include trials with a non-exercise control group. Acceptable control conditions include usual care (standard medical treatment), waiting list, health education, or no intervention. Trials comparing one form of exercise directly against another form of exercise without a non-exercise control arm will be excluded.

Study designs to be included Randomized controlled trials (RCTs). Cluster-randomized trials will be eligible if appropriate statistical adjustments are reported. Quasi-randomized or non-randomized trials will be excluded.

Eligibility criteria
Inclusion Criteria:

Study design: Randomized controlled trials (RCTs).

Participants: Adults (≥18 years) with diagnosed Rheumatoid Arthritis.

Intervention: Structured exercise programs (conventional or mind-body) exceeding routine daily activity.

Comparison: Non-exercise control (e.g., usual care, wait-list).

Outcomes: At least one of the following: depression, anxiety, or health-related quality of life.

Exclusion Criteria:

Studies evaluating multimodal programs where the effect of exercise cannot be isolated (e.g., exercise combined with psychotherapy vs. usual care).

Studies comparing two different exercise modalities without a non-exercise control.

Studies involving mixed populations (e.g., RA mixed with Osteoarthritis) unless data for RA participants are reported separately. There are no restrictions on publication language or date.

Information sources Electronic databases to be searched include PubMed, Embase, Cochrane Central Register of Controlled Trials (CENTRAL), Web of Science, Scopus, PsycINFO, and EBSCOhost. The search will cover the period from database inception to November 2025. In addition to electronic database searches, the reference lists of included studies and relevant systematic reviews will be screened manually. Forward citation tracking will also be conducted to identify further eligible trials.

Main outcome(s) The primary outcomes are depressive symptoms, anxiety, and health-related quality of life. These will be assessed using validated instruments reported in the included trials (e.g., HADS, BDI, SF-36, EQ-5D). Timing:

Xiao et al. INPLASY protocol 202610082. doi:10.37766/inplasy2026.1.0082 Downloaded from https://inplasy.com/inplasy-2026-1-0082/

Data will be extracted at the earliest post-intervention time point to improve comparability across trials. Effect measure: Continuous outcomes will be synthesized using Standardized Mean Differences (SMDs) with 95% confidence intervals.

Additional outcome(s) Safety outcomes, including adverse events and dropout rates (attrition), will be extracted to assess the feasibility and safety of the interventions. These will be summarized descriptively or quantitatively where data permit.

Data management Selection: All records will be imported into EndNote for deduplication. Two reviewers will independently screen titles and abstracts. Full-text reports of potentially eligible studies will be retrieved and assessed independently by two reviewers against eligibility criteria.
Extraction: Data extraction will be performed independently by two reviewers using a standardized form. Extracted data will include study characteristics, participant demographics, intervention details (using the FITT framework), and outcomes.
Resolution: Disagreements at screening or extraction stages will be resolved through discussion, with adjudication by a third reviewer if consensus cannot be reached.

Quality assessment / Risk of bias analysis Risk of bias will be assessed using the Cochrane Risk of Bias tool for randomized trials (RoB 1). Two reviewers will independently judge each domain as low, high, or unclear risk. Domains assessed will include:
Random sequence generation
Allocation concealment
Blinding of participants and personnel
Blinding of outcome assessment
Incomplete outcome data
Selective reporting
Other potential sources of bias Disagreements will be resolved by consensus or third-party adjudication.

Strategy of data synthesis We plan to perform a quantitative synthesis (meta-analysis) for the outcomes of depression, anxiety, and health-related quality of life.
Model: Random-effects meta-analysis using the inverse-variance method will be employed to account for potential between-study heterogeneity.
Effect Measure: Continuous outcomes will be synthesized as Standardized Mean Differences (SMDs, Hedges' g) with 95% confidence intervals (CIs).
Heterogeneity: Between-study heterogeneity will be assessed using the I^2 statistic and τ^2. If quantitative synthesis is not appropriate due to significant clinical or methodological diversity, a narrative synthesis will be provided.

Subgroup analysis Prespecified subgroup analyses will be conducted based on the type of exercise intervention to explore potential sources of heterogeneity and compare efficacy:
Mind-body exercise: e.g., Tai Chi, Yoga, Qigong, Yi Jin Jing.
Conventional exercise: e.g., aerobic training, resistance training, combined programs. Tests for subgroup differences will be performed to determine if the treatment effects vary significantly between these modalities.

Sensitivity analysis Sensitivity analyses will be performed to assess the robustness of the pooled results. A "leave-one-out" method will be used, where each study is iteratively removed from the analysis to observe whether omitting any single trial significantly alters the overall effect estimate or heterogeneity.

Language restriction No restrictions.

Country(ies) involved China.

Keywords Rheumatoid arthritis; Exercise; Depression; Anxiety; Health-related quality of life; Mind-body therapies; Systematic review; Meta-analysis.

Contributions of each author
Author 1 - xiao yan.
Author 2 - zhong jinghui.
Author 3 - yang xuesong.
Author 4 - zhang kebao.

Xiao et al. INPLASY protocol 202610082. doi:10.37766/inplasy2026.1.0082 Downloaded from https://inplasy.com/inplasy-2026-1-0082/
