# Supplementary material for: Effectiveness of exercise-based rehabilitation for depressive symptoms, anxiety, and health-related quality of life in adults with rheumatoid arthritis: A systematic review and meta-analysis of randomized controlled trials
Source: PLoS One. 2026 Jul 15;21(7):e0352173. doi: 10.1371/journal.pone.0352173 (PMC13372145; doi:10.1371/journal.pone.0352173)
Supplement: Attachment — Submitted filename: Response Letter .docx [file pone.0352173.s006.docx]

**Response to Editorial Office Requirements and Reviewers' Comments**

**Manuscript title:** Effectiveness of exercise-based rehabilitation for depressive symptoms, anxiety, and health-related quality of life in adults with rheumatoid arthritis: A systematic review and meta-analysis of randomized controlled trials

**Journal:** PLOS ONE

**Response format note:** Editorial Office and reviewer comments are reproduced in shaded boxes. Our responses are shown in blue, concrete manuscript changes are shown in red, and the corresponding locations in the revised manuscript are provided after each response.

## **Opening Statement**

We sincerely thank the Editorial Office and both reviewers for their careful evaluation of our manuscript and for the constructive technical, methodological, and interpretive comments. We have treated the revision as an opportunity to improve the transparency, reporting quality, and clinical interpretability of the manuscript. In response to the Editorial Office requirements, we checked the submission files, file naming, figure upload, main-text tables, Data Availability Statement, ORCID validation, and Supporting Information captions, and added the completed data extraction table as S2 Table. In response to Reviewer 1, we substantially reorganized the Introduction, expanded the Methods, clarified clinical heterogeneity and outcome harmonization, strengthened the Results, and shortened and refocused the Discussion and Conclusions. In response to Reviewer 2, we uploaded and cross-checked the Supporting Information files, adopted the Cochrane Risk of Bias 2 tool, and carefully considered the feasibility of a network meta-analysis. The point-by-point responses below describe exactly how each comment was addressed and where the corresponding revisions can be found in the revised manuscript.

## **Summary of Major Revisions**

| **Main revision** | **What changed** | **Where to check** |
| --- | --- | --- |
| Editorial Office requirements | Checked PLOS ONE style and file naming; updated the Data Availability Statement; validated the corresponding author ORCID; updated, cited, and uploaded Figure 5 to match the complete 7-trial QoL/HRQoL analysis; embedded main-text tables; uploaded Supporting Information files separately; and added consistent Supporting Information captions. | Submission package; revised manuscript; Data Availability Statement; Figure 5; Table 1; Table 2; S2 Table; Supporting Information captions. |
| Reviewer 1 -Introduction | Shortened and reorganized the Introduction, removed repetitive background and overly broad theory, added a clinically relevant fear-avoidance/kinesiophobia rationale, and clarified that the review primarily evaluates structured exercise overall while secondarily examining conventional versus mind-body exercise when data allow. | Revised manuscript, Introduction. |
| Reviewer 1 - Methods | Highlighted PRISMA 2020 more prominently, clarified protocol registration and deviations, refined eligibility and exclusion criteria, defined structured exercise, distinguished conventional and mind-body exercise, added outcome harmonization, adopted RoB 2, clarified publication-bias/small-study-effect assessment, and explained why additional subgroup analyses/meta-regression were not feasible. | Revised manuscript, Methods: Protocol and reporting; Search strategy; Eligibility criteria; Study selection and data extraction; Outcome harmonization; Risk of bias and certainty of evidence; Statistical analysis. |
| Reviewer 1 - Results | Expanded the description of clinical heterogeneity, self-reported outcomes, small sample sizes, MCID limitations, standardized effect interpretation, funnel-plot limitations, adverse-event reporting, and GRADE certainty. The Results now present the findings with more caution and more direct clinical context. | Revised manuscript, Results; Table 1; Table 2; Fig 2-Fig 6. |
| Reviewer 1 - Discussion and Conclusions | Shortened repetitive discussion, removed overextended speculative mechanisms, refocused interpretation on the review findings, clarified that mechanisms are hypotheses, and added practical but cautious clinical implications regarding individualized exercise selection and adjunctive use in comprehensive RA care. | Revised manuscript, Discussion and Conclusions. |
| Reviewer 2 - Supporting Information files | Uploaded and cross-checked the Supporting Information files, including the PRISMA 2020 checklist, complete search strategy, completed data extraction table, and registered INPLASY protocol. Added Supporting Information captions and matched in-text citations to the uploaded files. | Submission package; revised manuscript, Methods and Supporting Information captions; S1 Checklist; S1 Table; S2 Table; S1 Protocol. |
| Reviewer 2 - Risk of bias and network meta-analysis | Adopted the Cochrane RoB 2 tool for outcome-level risk-of-bias assessment in randomized trials. Considered network meta-analysis, but explained that the evidence network was too sparse and heterogeneous for reliable indirect comparisons; therefore, pairwise random-effects meta-analysis and modality subgroup analyses were retained. | Revised manuscript, Methods: Risk of bias and certainty of evidence; Statistical analysis; Results: Risk of bias and subgroup analyses; Discussion and Conclusions. |

# **Response to Editorial Office Requirements**

We thank the Editorial Office for the detailed technical and formatting requirements. We checked each item against the revised manuscript and the submission package so that the resubmitted files are internally consistent and ready for production review.

**Editorial requirement 1:** *Please ensure that your manuscript meets PLOS ONE's style requirements, including those for file naming.*

**Response:** Thank you for your reminder. We have carefully re-checked the revised manuscript and all submission files according to the PLOS ONE formatting and file preparation requirements. In particular, we reviewed the manuscript file, figures, tables, supporting information files, and file naming conventions to ensure that all uploaded materials are consistent and can be processed efficiently by the editorial office and production team. All figures have been revised according to the PLOS ONE figure guidelines (https://journals.plos.org/plosone/s/figures) and checked using the NAAS tool provided by PLOS. We confirmed that the figures meet the journal’s technical requirements regarding resolution, dimensions, file format, and other relevant specifications.

**Concrete revisions made:** We organized the manuscript file, individual figure files, in-text tables, and supporting information files in a consistent manner. The figure files have been provided separately and named according to their citations in the main text. We also avoided unnecessary special characters in file names and checked that the file names are consistent with the corresponding labels in the manuscript.

**Where to check in the revised manuscript:** Submission package; revised manuscript; figure files; Supporting Information files.

**Author action before resubmission:** Before resubmission, we will perform one final check that all uploaded file names match the labels used in the manuscript and online submission system.

**Editorial requirement 2:** *PLOS journals require all data underlying the findings described in the manuscript to be freely available to other researchers.*

**Response: Thank you for this important clarification. We revised the Data Availability Statement and updated the online submission form to comply with PLOS data-availability requirements. Because the present study is a systematic review and meta-analysis, no new individual participant-level dataset was generated. The data underlying the findings consist of the information extracted from the included published trials and the materials provided in the manuscript and Supporting Information files, including the completed data extraction table provided as S2 Table.**

**Concrete revisions made: We revised the Data Availability Statement to state clearly that all relevant data underlying the findings are included in the article and its Supporting Information files, including the completed data extraction table (S2 Table). This wording is intended to make the data source transparent and to avoid suggesting that an additional unpublished individual-level dataset exists.**

**Revised text excerpt:**

All relevant data underlying the findings of this systematic review and meta-analysis are included in the article and its Supporting Information files, including the completed data extraction table (S2 Table).

**Where to check in the revised manuscript: Revised manuscript, Data Availability Statement; S2 Table; online submission form.**

**Editorial requirement 3:** *Please ensure that the corresponding author has an ORCID iD validated in Editorial Manager.*

**Response:** Thank you for this instruction. The corresponding author has an ORCID iD, and the ORCID iD has been validated in Editorial Manager through the Fetch/Validate link in the ORCID field. We understand that this validation is required for the submission record and have completed it in the submission system.

**Concrete revisions made:** The corresponding-author ORCID validation was completed in Editorial Manager. No change to the scientific content of the manuscript was required for this item.

**Where to check in the revised manuscript:** Editorial Manager submission form.

**Editorial requirement 4:** *Please ensure that you refer to Figure 5 in your text as, if accepted, production will need this reference to link the reader to the figure.*

**Response:** Thank you for noting this issue. We retained Fig 5 as part of the submission, updated it to correspond to the complete 7-trial QoL/HRQoL analysis, and checked that it is cited in the main text. The figure is now referred to in the Results subsection reporting the QoL/HRQoL analysis and the conventional-versus-mind-body subgroup comparison, so the production team will be able to link the in-text citation to the figure if the manuscript is accepted.

**Concrete revisions made:** The Results now cite Fig 5 in the QoL/HRQoL analysis paragraph and report the updated 7-trial synthesis. The revised text reports 7 trials involving 564 participants, SMD = 0.45 (95% CI, 0.18 to 0.71), P = 0.001, I² = 50%, and no statistically significant subgroup difference between conventional and mind-body exercise.

**Revised text excerpt:**

Seven trials involving 564 participants reported quality-of-life or health-related quality-of-life (QoL/HRQoL) outcomes and were included in the quantitative synthesis. In a random-effects meta-analysis, exercise was associated with significantly improved QoL/HRQoL compared with control conditions, expressed as Hedges’ g standardized mean difference (SMD = 0.45; 95% CI, 0.18 to 0.71; P = 0.001). Statistical heterogeneity was moderate (I² = 50%). No statistically significant subgroup difference was observed between conventional exercise and mind-body exercise for QoL/HRQoL (Chi² = 0.26; P = 0.61) (Fig 5).

**Where to check in the revised manuscript:** Revised manuscript, Results, “Effects on health-related quality of life” subsection.

**Editorial requirement 5:** *Please upload a copy of Figure 5, to which you refer in your text. If the figure is no longer to be included as part of the submission, please remove all reference to it within the text.*

**Response:** Thank you for this reminder. Fig 5 remains part of the submission, and we uploaded the updated figure file with a file name matching the in-text figure citation. We also cross-checked the figure content against the revised Results text to ensure that both now report the complete 7-trial QoL/HRQoL synthesis and the same modality subgroup comparison.

**Concrete revisions made:** Fig 5 now presents the random-effects meta-analysis for QoL/HRQoL based on 7 trials (564 participants) and the subgroup comparison between conventional exercise and mind-body exercise. We retained the in-text citation to Fig 5 and uploaded the corresponding separate figure file. The previous 4-trial analysis is no longer described as the main HRQoL result; if retained, it is treated only as an exploratory instrument-based supplementary analysis.

**Where to check in the revised manuscript:** Submission package; revised manuscript, Results, Fig 5 citation; separate Fig 5 file.

**Editorial requirement 6:** *Please include your tables as part of your main manuscript and remove the individual files. Supplementary tables should be uploaded separately as Supporting Information files.*

**Response:** Thank you for this guidance. We revised the submission package so that main-text tables and supplementary tables are handled differently, in accordance with the journal requirements. Tables that are part of the main article are included directly in the manuscript file, whereas supplementary material is uploaded separately as Supporting Information.

**Concrete revisions made: Table 1 and Table 2 have been included directly in the main manuscript file. Table 1 summarizes the characteristics of included studies and exercise interventions, and Table 2 presents the GRADE summary of evidence quality and effect estimates. The supplementary search strategy and completed data extraction table are uploaded separately as Supporting Information files and are cited as S1 Table and S2 Table, respectively.**

**Where to check in the revised manuscript: Revised manuscript, Table 1 and Table 2; Supporting Information files S1 Table and S2 Table; Supporting Information captions.**

**Editorial requirement 7:** *Please include captions for your Supporting Information files at the end of your manuscript, and update any in-text citations to match accordingly.*

**Response:** Thank you for this comment. We revised the Supporting Information captions at the end of the manuscript and checked the in-text citations for consistency. We also checked that each Supporting Information item mentioned in the manuscript corresponds to a file uploaded in the submission package.

**Concrete revisions made: The Supporting Information files are now labeled consistently as S1 Checklist, S1 Table, S2 Table, and S1 Protocol. The Methods section cites the PRISMA checklist, protocol, and completed data extraction table; the Search strategy section cites the complete search strategy; and the manuscript-ending captions match the uploaded file labels.**

**Revised text excerpt:**

S1 Checklist. PRISMA 2020 checklist.
S1 Table. Search strategy.
S2 Table. Data extraction table.
S1 Protocol. Registered INPLASY protocol.

**Where to check in the revised manuscript: Revised manuscript, Methods, “Protocol and reporting,” “Search strategy,” and “Study selection and data extraction” subsections; Supporting Information captions at the end of the manuscript.**

**Author action before resubmission: We also corrected the manuscript-ending protocol caption so that it consistently reads “S1 Protocol” and added “S2 Table. Data extraction table.” so that the manuscript captions match the response letter and submission files.**

**Editorial requirement 8:** *If the reviewer comments include a recommendation to cite specific previously published works, please review and evaluate these publications to determine whether they are relevant and should be cited.*

**Response:** Thank you for this guidance. We reviewed the suggested and newly relevant publications carefully and added citations only when they directly supported the revised rationale, methods, interpretation, or reporting standards. We did not add citations simply because they were suggested; instead, we evaluated whether each citation strengthened the scientific argument and was relevant to the revised manuscript.

**Concrete revisions made:** Relevant references were added or retained where they supported fear-avoidance/kinesiophobia, PRISMA 2020 reporting, structured exercise definitions, mind-body exercise classification, RoB 2, GRADE, effect-size calculation, clinically important differences, and intervention-reporting frameworks. Citations not directly relevant to the manuscript were not added solely for formal reasons.

**Where to check in the revised manuscript:** Revised manuscript, Introduction, Methods, Discussion, Conclusions, and References.

# **Response to Reviewer 1**

We sincerely thank Reviewer 1 for the careful and constructive evaluation of our manuscript. The comments helped us improve the focus of the Introduction, the transparency of the Methods, the cautious interpretation of the Results, and the clinical usefulness of the Discussion and Conclusions. We have revised the manuscript point by point as described below.

## **Reviewer 1 - Introduction**

**Comment 1.1:** *The introduction is relatively lengthy and somewhat repetitive in several sections.*

**Response:** Thank you for this valuable comment. We agree that the original Introduction was longer than necessary and contained repeated background information. In the revised manuscript, we shortened and reorganized the Introduction so that it moves more directly from the clinical burden of rheumatoid arthritis to the rationale for evaluating depressive symptoms, anxiety, and health-related quality of life as patient-centered outcomes.

**Concrete revisions made:** We removed or merged repetitive statements on RA disease burden, psychological symptoms, rehabilitation relevance, and the broader exercise literature. We retained only the background needed to support the research question: RA is associated with pain, fatigue, disability, psychological distress, and impaired HRQoL; exercise is recommended as part of comprehensive inflammatory arthritis management; and previous reviews have not fully clarified the effects of exercise on depressive symptoms, anxiety, and HRQoL in adults with RA.

**Revised text excerpt:**

Rheumatoid arthritis (RA) is a chronic systemic autoimmune disease characterized by synovial inflammation, pain, fatigue, progressive joint damage, and functional limitation. Beyond inflammatory and structural manifestations, RA is also accompanied by a considerable psychological burden.

**Where to check in the revised manuscript:** Revised manuscript, Introduction.

**Comment 1.2:** *Some theoretical explanations are overly extensive and delay the presentation of the primary research objective.*

**Response:** Thank you for pointing out this issue. We agree that several theoretical explanations in the original Introduction were too broad and delayed the presentation of the review objective. We therefore reduced extended background discussion and made the final research objective more prominent and easier to identify.

**Concrete revisions made:** We deleted or condensed long theoretical sections on potential exercise mechanisms, pharmacological management, disease-activity monitoring, rehabilitation delivery contexts, and implementation-oriented exercise research. The revised Introduction now focuses on the clinical uncertainty that directly motivates this review and ends with a concise statement of the objective.

**Revised text excerpt:**

Accordingly, this systematic review and meta-analysis aimed to evaluate the effects of structured exercise interventions on depressive symptoms, anxiety, and HRQoL in adults with RA. Where sufficient data were available, we further examined whether these effects differed between conventional exercise and mind-body exercise interventions.

**Where to check in the revised manuscript:** Revised manuscript, Introduction, final paragraph.

**Comment 1.3:** *It remains unclear whether the authors intended to evaluate exercise interventions broadly or compare the effectiveness of different exercise modalities.*

**Response:** Thank you for identifying this lack of clarity. We agree that the original Introduction did not sufficiently explain the relationship between the overall evaluation of structured exercise interventions and the comparison of different exercise modalities. In the revised manuscript, we clarified that the primary aim was to evaluate structured exercise interventions as a broad rehabilitation category, while the comparison between conventional exercise and mind-body exercise was a secondary analysis performed only when data were sufficient.

**Concrete revisions made:** We revised the final paragraph of the Introduction and aligned it with the Methods. The manuscript now explains that structured exercise includes both conventional exercise and mind-body exercise. It also clarifies that subgroup analyses were intended to explore whether effects differed between these two broad categories, rather than to claim definitive superiority of one specific exercise modality over another.

**Revised text excerpt:**

Exercise interventions vary in modality, ranging from conventional exercise such as resistance training and aerobic exercise to mind-body approaches such as Tai Chi and yoga. Therefore, a focused synthesis of depressive symptoms, anxiety, and HRQoL, with further consideration of differences between conventional and mind-body exercise, may help clarify the role of exercise in patient-centered RA management.

**Where to check in the revised manuscript:** Revised manuscript, Introduction, paragraphs 3-4; Methods, “Eligibility criteria” and “Statistical analysis” subsections.

**Suggestion 1.4:** *The authors may consider discussing fear-avoidance behavior or kinesiophobia in RA patients, as this is highly relevant to exercise adherence and rehabilitation participation.*

**Response:** We sincerely thank the reviewer for this clinically important suggestion. We fully agree that pain-related fear, reduced confidence in movement, and concern about symptom exacerbation are highly relevant to exercise participation and rehabilitation adherence in RA. This suggestion helped us strengthen the clinical rationale for focusing on psychological outcomes and HRQoL rather than treating exercise only as a physical-function intervention.

**Concrete revisions made:** We added discussion of fear-avoidance beliefs and kinesiophobia to the Introduction. We now explain that exercise participation in RA may be influenced not only by disease activity and physical capacity, but also by pain-related beliefs, confidence in movement, and fear of symptom exacerbation. We also added RA-specific evidence showing associations between fear-avoidance/kinesiophobia and pain, functional status, depressive symptoms, and quality of life.

**Revised text excerpt:**

This question is clinically important because exercise participation in RA may be shaped not only by physical capacity and disease activity, but also by pain-related beliefs, confidence in movement, and fear of symptom exacerbation. The fear-avoidance model suggests that pain-related fear may lead to activity avoidance, physical disuse, and disability in chronic musculoskeletal conditions. In RA-specific studies, fear-avoidance beliefs and kinesiophobia-two related forms of pain-related fear-have been associated with greater pain, poorer functional status, depressive symptoms, and reduced quality of life.

**Where to check in the revised manuscript:** Revised manuscript, Introduction, paragraph 2.

## **Reviewer 1 - Methods**

**Comment 2.1:** *The manuscript should more explicitly emphasize adherence to the PRISMA 2020 guideline. Although PRISMA was mentioned, this reporting framework should be highlighted more prominently as the methodological standard.*

**Response:** Thank you for this valuable comment. We agree that the original Methods mentioned PRISMA but did not sufficiently emphasize PRISMA 2020 as the main methodological reporting framework. We therefore revised the “Protocol and reporting” subsection so that adherence to PRISMA 2020 is stated at the beginning of the Methods and connected to the checklist provided as Supporting Information.

**Concrete revisions made:** We now state explicitly that the systematic review and meta-analysis was conducted and reported in accordance with the PRISMA 2020 statement. We also added that the completed PRISMA 2020 checklist is provided as S1 Checklist, clarified that the review protocol was registered with INPLASY and provided as S1 Protocol, and reported protocol deviations transparently, including the English-language restriction and adoption of RoB 2.

**Revised text excerpt:**

This systematic review and meta-analysis was conducted and reported in accordance with the PRISMA 2020 statement. The completed PRISMA 2020 checklist is provided as S1 Checklist. The review protocol was retrospectively registered with INPLASY (INPLASY202610082) after completion of the database search and is provided as S1 Protocol.

**Where to check in the revised manuscript:** Revised manuscript, Methods, “Protocol and reporting” subsection; S1 Checklist; S1 Protocol.

**Comment 2.2:** *The inclusion criteria regarding exercise interventions were very broad. The review combined aerobic exercise, resistance training, walking programs, yoga, Tai Chi, and Yi Jin Jing into pooled analyses, which may substantially increase clinical heterogeneity.*

**Response:** Thank you for raising this important methodological concern. We agree that the included exercise interventions are clinically heterogeneous. We also agree that this heterogeneity must be made explicit so that readers do not interpret the pooled effects as representing one specific exercise prescription. In the revised manuscript, we clarified the definition of structured exercise, separated conventional and mind-body exercise categories, and interpreted pooled estimates as average effects across a heterogeneous rehabilitation evidence base.

**Concrete revisions made:** We revised the Eligibility criteria section to define structured exercise as planned, repeated physical activity or therapeutic movement intended to improve or maintain physical fitness, physical function, symptoms, or health. Eligible interventions had to include an active movement component and at least one exercise descriptor. We then distinguished conventional exercise from mind-body exercise in the Methods and summarized intervention features in Table 1. To address heterogeneity analytically, we used random-effects models and performed planned subgroup analyses by intervention category when sufficient studies were available.

**Revised text excerpt:**

Structured exercise was defined as planned, repeated physical activity or therapeutic movement intended to improve or maintain physical fitness, physical function, symptoms, or health. Eligible interventions had to include an active movement component and report at least one exercise descriptor, such as exercise type, frequency, session duration, intensity, supervision, progression, or total intervention duration. Conventional exercise included aerobic, resistance, strengthening, flexibility, balance, dance-based, joint-specific, water-based, or mixed exercise programs. Mind-body exercise included movement-based practices such as Tai Chi, Yoga, Qigong, Ba Duan Jin, or Yi Jin Jing.

**Where to check in the revised manuscript:** Revised manuscript, Methods, “Eligibility criteria” and “Statistical analysis” subsections; Table 1; Discussion.

**Comment 2.3:** *The exclusion criteria were insufficiently described. It was unclear whether non-English studies were excluded, whether grey literature was included, whether conference abstracts were considered, whether patients with severe psychiatric disorders were excluded, or whether co-interventions were allowed.*

**Response:** Thank you for pointing out that the exclusion criteria required more detail. We revised both the Search strategy and Eligibility criteria sections to clarify language restrictions, grey literature, conference-only reports, severe psychiatric disorders, and multimodal or co-intervention issues. We also reported the language restriction as a deviation from the registered protocol and acknowledged its potential influence as a limitation.

**Concrete revisions made:** The revised Methods now state that only English-language full-text articles were included, grey literature was not systematically searched, and conference-only reports without sufficient outcome data were excluded. We expanded the exclusion criteria to include non-randomized or non-original designs, studies without separately extractable RA data, interventions without active movement, multimodal interventions where the independent effect of exercise could not be isolated, comparisons without a non-exercise control arm, and studies without eligible validated patient-reported outcomes. We also clarified that we did not apply an additional review-level exclusion criterion for severe psychiatric disorders unless such criteria were specified in the original trial.

**Revised text excerpt:**

Our exclusion criteria were as follows: (1) non-randomized, quasi-randomized, observational, cross-sectional, case-series, protocol-only, or conference-only reports without sufficient outcome data; (2) studies without RA participants, or studies including mixed rheumatic, musculoskeletal, or inflammatory populations without separately extractable RA data; (3) interventions without an active movement component; (4) multimodal interventions in which the independent effect of exercise could not be isolated; (5) comparisons of one exercise intervention only with another exercise intervention without a non-exercise control arm; and (6) studies that did not report eligible psychological or HRQoL outcomes using a validated patient-reported measure.

**Where to check in the revised manuscript:** Revised manuscript, Methods, “Protocol and reporting,” “Search strategy,” and “Eligibility criteria” subsections.

**Comment 2.4:** *The manuscript did not sufficiently explain how different psychological instruments were harmonized beyond the use of standardized mean differences.*

**Response:** Thank you for raising this important methodological point. We agree that the original manuscript did not sufficiently explain how different instruments were harmonized before synthesis. Standardized mean differences address differences in scale units, but harmonization also requires consistent outcome-domain classification, avoidance of duplicate within-study contributions, and standardization of effect direction. We therefore added a dedicated “Outcome harmonization” subsection.

**Concrete revisions made:** We now specify that eligible instruments were classified before synthesis as assessing depressive symptoms, anxiety, or global/summary HRQoL. If a study reported multiple instruments for the same outcome domain, we retained one validated and most domain-specific measure to avoid counting the same study more than once for the same outcome. For HRQoL, global or summary measures were prioritized over isolated subscales. We also clarified the direction of effects before pooling.

**Revised text excerpt:**

Before synthesis, eligible instruments were classified as assessing depressive symptoms, anxiety, or global/summary HRQoL. If multiple instruments were reported for the same domain within a study, one validated and most domain-specific measure was retained. Global or summary HRQoL measures were prioritized over isolated subscales. Effect directions were harmonized before pooling so that negative SMDs indicated lower depressive symptoms or anxiety, whereas positive SMDs indicated better HRQoL.

**Where to check in the revised manuscript:** Revised manuscript, Methods, “Outcome harmonization” subsection; Methods, “Statistical analysis” subsection.

**Comment 2.5:** *Publication bias and small-study effects were only briefly discussed.*

**Response:** Thank you for this valuable comment. We agree that publication bias and small-study effects required a more cautious and explicit treatment. We therefore expanded the Methods and Results to describe how small-study effects were assessed and to explain the limitations of funnel-plot interpretation when only a small number of trials contributed to each outcome.

**Concrete revisions made:** The revised Methods now state that small-study effects and potential publication bias were assessed separately for each outcome, with visual inspection of funnel plots when at least 10 studies were available. Formal asymmetry tests were not performed because the number of studies was small and only depressive symptoms reached the conventional threshold for funnel-plot assessment. The revised Results now report that the depression funnel plot did not suggest marked asymmetry, but small-study effects and publication bias could not be excluded. Anxiety and HRQoL funnel plots are presented descriptively only because fewer than 10 studies contributed to those outcomes.

**Revised text excerpt:**

Small-study effects and potential publication bias were assessed for each outcome. Funnel plots were visually inspected when at least 10 studies were available for a given outcome. Formal tests for funnel plot asymmetry were not performed because the number of studies remained small, with only one outcome reaching the conventional threshold for funnel plot assessment. For outcomes with fewer than 10 studies, publication bias was considered qualitatively and incorporated into the GRADE assessment where relevant.

**Where to check in the revised manuscript:** Revised manuscript, Methods, “Statistical analysis” subsection; Results, “Small-study effects, adverse events, and certainty of evidence” subsection; Fig 6; Table 2.

**Suggestion 2.6:** *Additional subgroup analyses based on exercise intensity, supervision, intervention duration, disease activity, and baseline depression severity may improve clinical interpretability.*

**Response:** Thank you for this clinically valuable suggestion. We agree that these variables may influence the effect of exercise on depressive symptoms, anxiety, and HRQoL, and that such subgroup analyses would be clinically informative if the evidence base were sufficiently large and consistently reported. We carefully considered these analyses but concluded that performing them would not be statistically reliable or clinically interpretable in the current review.

**Concrete revisions made:** We added a clearer explanation that these variables were extracted descriptively when reported, but were not examined as formal effect modifiers because the number of studies per outcome was limited and covariates such as intensity, supervision, disease activity, and baseline depression severity were inconsistently reported. Further stratification would have created very small subgroups and unstable estimates. We therefore retained the prespecified conventional-versus-mind-body subgroup comparison, reported Table 1 to aid clinical interpretation, and discussed the need for future trials with better intervention reporting.

**Revised text excerpt:**

Available details, including exercise type, intervention duration, frequency, were extracted and are reported descriptively. These variables were used descriptively and were not examined as formal effect modifiers because the number of studies available for each outcome was limited. Meta-regression was not conducted because the number of studies per outcome was insufficient and relevant covariates were inconsistently reported.

**Where to check in the revised manuscript:** Revised manuscript, Methods, “Study selection and data extraction” and “Statistical analysis” subsections; Results, Table 1; Discussion, limitations and future research paragraphs.

## **Reviewer 1 - Results**

**Comment 3.1:** *Clinical heterogeneity was substantial regarding exercise modality, intervention duration, frequency, comparator groups, and outcome instruments.*

**Response:** Thank you for highlighting this important issue. We agree that clinical and measurement heterogeneity are central considerations in interpreting this review. We therefore expanded the Results to describe the heterogeneity of participants, intervention modality, dose, delivery setting, supervision, comparator conditions, and outcome instruments before presenting the pooled effects.

**Concrete revisions made:** The revised Results now describe the range of intervention duration, frequency, exercise categories, comparator types, and outcome instruments. We also explicitly state that the included trials were clinically heterogeneous and that pooled estimates should be interpreted as average effects of structured exercise programs rather than evidence for a single optimal exercise prescription.

**Revised text excerpt:**

The included trials were clinically heterogeneous in population characteristics, exercise modality, intervention dose, delivery setting, supervision, and comparator type. Several trials were small, with six trials enrolling fewer than 60 participants in total, which should be considered when interpreting the precision and robustness of the pooled estimates.

**Where to check in the revised manuscript:** Revised manuscript, Results, “Study and participant characteristics” subsection; Table 1; Discussion.

**Comment 3.2:** *Most outcomes relied on self-reported questionnaires, increasing susceptibility to expectancy bias.*

**Response:** Thank you for emphasizing this important source of bias. We agree that depressive symptoms, anxiety, and HRQoL were mainly measured using self-reported questionnaires and that exercise trials often cannot blind participants or intervention personnel. These issues are now discussed more explicitly in relation to risk of bias and certainty of evidence.

**Concrete revisions made:** We revised the Risk of bias Results to explain that lack of blinding and self-reported outcomes may increase expectancy or response bias. We also incorporated these concerns into the GRADE assessment, where risk of bias was one reason for downgrading evidence certainty across outcomes.

**Revised text excerpt:**

Because exercise interventions are behavioral in nature, blinding of participants and intervention personnel was generally infeasible. In addition, depressive symptoms, anxiety, and HRQoL were mainly assessed using self-reported questionnaires, which may have increased the risk of expectancy or response bias.

**Where to check in the revised manuscript:** Revised manuscript, Results, “Risk of bias” and “Small-study effects, adverse events, and certainty of evidence” subsections; Discussion, limitations paragraph; Table 2.

**Comment 3.3:** *Several included studies had relatively small sample sizes (<60 participants).*

**Response:** Thank you for pointing out this important issue. We agree that several included trials were small and that this affects the precision and robustness of the pooled estimates. We now state this more clearly in the Results and reflect it in the certainty-of-evidence assessment.

**Concrete revisions made:** We added an explicit statement that six of the 15 included trials enrolled fewer than 60 participants in total. We also discussed imprecision due to small sample sizes and few contributing trials, particularly for anxiety and HRQoL, in the GRADE assessment and Discussion.

**Revised text excerpt:**

Several trials were small, with six trials enrolling fewer than 60 participants in total, which should be considered when interpreting the precision and robustness of the pooled estimates. Using GRADE, the certainty of evidence was rated as moderate for depressive symptoms and low for anxiety and HRQoL. The main reasons for downgrading were risk of bias related to deviations from intended interventions and self-reported outcome measurement, imprecision due to small sample sizes and few contributing trials, and limited ability to assess publication bias.

**Where to check in the revised manuscript:** Revised manuscript, Results, “Study and participant characteristics” and “Small-study effects, adverse events, and certainty of evidence” subsections; Table 2; Discussion.

**Comment 3.4:** *Minimal clinically important differences (MCID) were not discussed.*

**Response:** Thank you for this important comment. We agree that MCID or minimally important difference thresholds are important for interpreting whether statistically significant standardized effects translate into clinically meaningful improvements for patients. We revised the Results and Discussion to explain why direct MCID-based interpretation was limited in this review.

**Concrete revisions made:** We now state that MCID thresholds or proportions of participants achieving clinically important improvement were not consistently reported across trials. We also added discussion explaining that SMDs were necessary because the included trials used different instruments, but SMDs are less intuitive for clinical decision-making. We therefore interpret the findings cautiously and recommend that future trials report the proportion of participants achieving clinically meaningful improvement when validated thresholds are available.

**Revised text excerpt:**

MCID thresholds or proportions of participants achieving clinically important improvement were not consistently reported across trials; therefore, pooled SMDs were interpreted as standardized effects rather than direct estimates of clinically important improvement.

**Where to check in the revised manuscript:** Revised manuscript, Results, “Study and participant characteristics” subsection; Discussion, clinical interpretation paragraph; Conclusions and future research statements.

**Suggestion 3.5:** *Consider adding a dedicated table summarizing FITT principles; additional analyses comparing supervised versus home-based exercise may be clinically valuable; reporting absolute effects alongside SMDs may improve interpretability for clinicians.*

**Response:** Thank you for these clinically valuable suggestions. We agree that FITT reporting, supervision comparisons, and clinically interpretable effect estimates would improve usefulness for clinicians. We considered each suggestion carefully and revised the manuscript to improve descriptive reporting, while avoiding analyses or absolute-effect conversions that the data could not support reliably.

**Concrete revisions made:** Rather than adding a separate FITT table, we expanded Table 1 to include the available intervention details, including type, frequency, duration, intensity, supervision, and other exercise-intervention details when reported. We did not conduct supervised-versus-home-based subgroup analyses because supervision and home-practice characteristics were inconsistently reported and the number of studies per outcome was limited. We also did not report absolute effects converted from SMDs because the primary studies used different outcome instruments with different scoring ranges and rarely reported MCID responder data. Instead, we clarified these limitations and recommended more complete reporting in future trials.

**Revised text excerpt:**

Detailed study characteristics and available intervention details, including type, frequency, duration, intensity, supervision, and other exercise-intervention details where reported, are summarized in Table 1. Standardized mean differences were appropriate because the included trials used different instruments to assess similar constructs, but they are less intuitive for clinical decision-making. Therefore, although the results support the potential benefits of exercise interventions, the extent to which these changes translate into patient-perceived clinical improvement requires further investigation.

**Where to check in the revised manuscript:** Revised manuscript, Table 1; Methods, “Study selection and data extraction” and “Statistical analysis” subsections; Results, “Study and participant characteristics” subsection; Discussion.

## **Reviewer 1 - Discussion**

**Comment 4.1:** *The discussion is overly long and occasionally repetitive.*

**Response:** Thank you for this valuable comment. We agree that the original Discussion was too long and sometimes repeated information already presented in the Introduction or Results. We therefore shortened and reorganized the Discussion to focus on interpretation of the main findings, certainty of evidence, clinical heterogeneity, measurement limitations, practical implications, and future research needs.

**Concrete revisions made:** We removed repetitive discussion of the general RA burden, broad rehabilitation background, and repeated summaries of exercise benefits. The revised Discussion now follows a clearer structure: summary of findings, relation to previous reviews, clinical interpretation of SMDs and HRQoL, heterogeneity and modality interpretation, plausible mechanisms with caution, limitations, and future directions.

**Where to check in the revised manuscript:** Revised manuscript, Discussion.

**Comment 4.2:** *Some mechanistic explanations are speculative and excessively broad.*

**Response:** Thank you for pointing this out. We agree that the original manuscript included broad mechanistic explanations that were not directly tested in the included trials. We therefore removed or shortened speculative mechanistic pathways and retained only clinically plausible explanations that are directly relevant to RA exercise rehabilitation and psychological outcomes.

**Concrete revisions made:** We removed extended discussion of neuroendocrine pathways, inflammatory signaling pathways, circadian dysregulation, neuroimmune mechanisms, and brain-derived neurotrophic factor signaling. The revised Discussion instead frames mechanisms as hypotheses, including improved physical function, sleep, symptom coping, self-efficacy, behavioral activation, confidence in movement, and reduced pain-related fear. We explicitly state that mechanistic variables were rarely measured directly and should not be interpreted as confirmed causal pathways.

**Revised text excerpt:**

Several plausible mechanisms may explain the observed benefits. Exercise may improve depressive symptoms and anxiety by enhancing physical function, sleep, symptom coping, self-efficacy, behavioral activation, and confidence in movement. However, mechanistic variables were rarely measured directly in the included trials. These explanations should therefore be regarded as hypotheses rather than confirmed causal pathways.

**Where to check in the revised manuscript:** Revised manuscript, Discussion, mechanistic interpretation paragraph.

**Comment 4.3:** *Certain sections resemble a narrative review rather than a focused interpretation of study findings.*

**Response:** Thank you for this constructive comment. We agree that some portions of the original Discussion read like a broad narrative review rather than a focused interpretation of the evidence synthesized in this manuscript. We revised the Discussion so that each paragraph is tied more directly to the findings, limitations, or implications of the present review.

**Concrete revisions made:** We reduced broad discussion of implementation research, digital exercise delivery, service-delivery models, and long-term rehabilitation pathways unless directly relevant to the present findings. The revised text now emphasizes what the current meta-analysis shows, what it does not show, why certainty is limited for anxiety and HRQoL, and what future studies should report to make the evidence more clinically interpretable.

**Revised text excerpt:**

Although the pooled effects were generally favorable, their clinical interpretation depends on the measurement instruments used and the availability of clinically meaningful thresholds. Standardized mean differences were appropriate because the included trials used different instruments to assess similar constructs, but they are less intuitive for clinical decision-making.

**Where to check in the revised manuscript:** Revised manuscript, Discussion.

## **Reviewer 1 - Conclusions**

**Comment 5.1:** *Practical recommendations for clinicians remain somewhat limited.*

**Response:** Thank you for this valuable comment. We agree that the original Conclusion summarized the meta-analytic findings but did not provide enough practical interpretation for clinicians. We revised the Conclusion to state more clearly how the findings may be used in comprehensive RA care while avoiding overstatement.

**Concrete revisions made:** The revised Conclusion now states that structured exercise may be considered an adjunctive component of comprehensive RA care, including for patients with persistent psychological distress, reduced activity confidence, limited participation, or impaired perceived health. We also clarify that exercise should not replace pharmacologic disease control or formal mental-health care when these are indicated. Finally, we add that exercise selection should be individualized based on symptoms, functional limitations, preferences, supervision access, home-practice feasibility, and safety.

**Revised text excerpt:**

These findings support structured exercise as an adjunctive component of comprehensive RA care, including for patients with persistent psychological distress, reduced activity confidence, limited participation, or impaired perceived health. Exercise should not be viewed as a substitute for pharmacologic disease control or formal mental-health care when these are indicated. Exercise selection should therefore be individualized according to symptoms, functional limitations, patient preferences, access to supervision, home-practice feasibility, and safety considerations.

**Where to check in the revised manuscript:** Revised manuscript, Conclusions.

# **Response to Reviewer 2**

We sincerely thank Reviewer 2 for the careful evaluation of our manuscript and for the helpful recommendations regarding Supporting Information files, risk-of-bias assessment, and the feasibility of network meta-analysis. These comments allowed us to improve the auditability of the submission package and strengthen the methodological transparency of the review.

**Comment 1:** *Missing Supporting Information files: The absence of the supplementary files/eTables limited assessment of methodological rigor and verification of information reported in the manuscript.*

**Response:** We sincerely thank the reviewer for this important comment, and we apologize that the Supporting Information files were not available for review during the previous submission. We recognize that these files are necessary for evaluating search transparency, PRISMA adherence, and protocol registration. We have therefore checked the revised submission package carefully and uploaded the relevant Supporting Information files.

**Concrete revisions made:** The revised submission includes the completed PRISMA 2020 checklist, the complete search strategy, and the registered INPLASY protocol. We also added captions at the end of the manuscript and checked the in-text citations so that the manuscript, response letter, and uploaded files use consistent labels. This should allow the reviewer and Editorial Office to verify the search strategy, reporting checklist, and protocol-related information directly.

**Revised text excerpt:**

S1 Checklist. PRISMA 2020 checklist.
S1 Table. Search strategy.
S2 Table. Data extraction table.
S1 Protocol. Registered INPLASY protocol.

**Where to check in the revised manuscript: Submission package; revised manuscript, Methods, “Protocol and reporting,” “Search strategy,” and “Study selection and data extraction” subsections; Supporting Information captions.**

**Author action before resubmission:** We will ensure that S1 Checklist, S1 Table, and S1 Protocol are all uploaded as separate Supporting Information files during resubmission and that their labels match the manuscript captions exactly.

**Comment 2:** *Risk-of-bias assessment tool: Consideration was requested for adopting the Cochrane RoB 2 tool instead of the original ROB tool, as RoB 2 may provide a more robust risk-of-bias assessment for randomized studies.*

**Response:** Thank you for this helpful suggestion. We agree that the Cochrane Risk of Bias 2 tool is more appropriate for randomized controlled trials and provides a more structured outcome-level assessment than the tool used in the original submission. We therefore revised the risk-of-bias assessment approach and updated the Methods, Results, and certainty-of-evidence interpretation accordingly.

**Concrete revisions made:** Risk of bias is now assessed at the outcome level using RoB 2 for randomized trials. Judgments were made for the effect of assignment to intervention and classified as low risk of bias, some concerns, or high risk of bias according to RoB 2 guidance. The Results now describe the main sources of concern, particularly deviations from intended interventions and measurement of self-reported outcomes. The GRADE assessment also reflects these risk-of-bias judgments.

**Revised text excerpt:**

Risk of bias was assessed at the outcome level using the Cochrane Risk of Bias 2 tool (RoB 2) for randomized trials. Risk-of-bias judgments were made for the effect of assignment to the intervention. The overall judgment for each outcome was classified as low risk of bias, some concerns, or high risk of bias according to RoB 2 guidance.

**Where to check in the revised manuscript:** Revised manuscript, Methods, “Risk of bias and certainty of evidence” subsection; Results, “Risk of bias” subsection; Fig 2; Table 2.

**Comment 3:** *Consideration of network meta-analysis: Given the heterogeneity of the included exercise interventions, consideration was requested regarding whether a network meta-analysis could be conducted to compare different intervention types.*

**Response:** Thank you for this thoughtful suggestion. We agree that a network meta-analysis could be very informative if there were a sufficiently large, well-connected, and consistently reported evidence network comparing different exercise modalities. We carefully considered whether such an analysis could be conducted in the present review. However, after examining the number of trials, intervention categories, comparators, and outcome-specific evidence structure, we concluded that a network meta-analysis would not be reliable for the current dataset.

**Concrete revisions made:** The available evidence was sparse and heterogeneous. Only 15 trials were included overall, and fewer trials contributed to each outcome. Specific modalities such as walking, aerobic/resistance exercise, yoga, Tai Chi, Yi Jin Jing, and multimodal exercise were often represented by one or very few studies. Most studies compared one exercise intervention with usual care, health education, counseling, or another non-exercise comparator, rather than directly comparing active exercise modalities. As a result, a network meta-analysis would have relied heavily on indirect comparisons across a weakly connected network, increasing the risk of unstable and potentially misleading estimates. We therefore retained pairwise random-effects meta-analysis and the prespecified subgroup comparison between conventional and mind-body exercise when sufficient data were available. We also made the interpretation more cautious and clarified that absence of subgroup differences should not be interpreted as equivalence among modalities.

**Revised text excerpt:**

The absence of statistically significant subgroup differences between conventional and mind-body exercise should not be interpreted as evidence of equivalence. Both categories included diverse interventions, and subgroup analyses were limited by small study numbers and within-category heterogeneity. Current evidence does not establish the superiority of any specific exercise modality or delivery format.

**Where to check in the revised manuscript:** Revised manuscript, Methods, “Statistical analysis” subsection; Results, subgroup analyses; Discussion, clinical heterogeneity paragraph; Conclusions.

## **Closing Statement**

We again sincerely thank the Editorial Office and both reviewers for their careful reading and constructive comments. We have revised the manuscript and submission package in detail, with particular attention to reporting transparency, methodological rigor, cautious interpretation, and clinical relevance. We believe that the revised manuscript is clearer, more transparent, and more clinically interpretable as a result of the reviewers’ recommendations. We hope that the revised manuscript and the detailed responses above adequately address all concerns raised during review.
