# Supplementary material for: Effectiveness of exercise-based rehabilitation for depressive symptoms, anxiety, and health-related quality of life in adults with rheumatoid arthritis: A systematic review and meta-analysis of randomized controlled trials
Source: PLoS One. 2026 Jul 15;21(7):e0352173. doi: 10.1371/journal.pone.0352173 (PMC13372145; doi:10.1371/journal.pone.0352173)
Supplement: Peer review history

# Peer review: Decision Letter 0

**DOI:** 10.1371/journal.pone.0352173.r001

**Authors:** Marwan Salih Al-Nimer

26 May 2026

PONE-D-26-20182

Effectiveness of Exercise-Based Rehabilitation for Depressive Symptoms, Anxiety, and Health-Related Quality of Life in Adults With Rheumatoid Arthritis: A Systematic Review and Meta-Analysis of Randomized Controlled Trials

PLOS One

Dear Dr. xiao,

Thank you for submitting your manuscript to PLOS ONE. After careful consideration, we feel that it has merit but does not fully meet PLOS ONE’s publication criteria as it currently stands. Therefore, we invite you to submit a revised version of the manuscript that addresses the points raised during the review process.

ACADEMIC EDITOR: Major revision

Please submit your revised manuscript by Jul 10 2026 11:59PM. If you will need more time than this to complete your revisions, please reply to this message or contact the journal office at plosone@plos.org. When you're ready to submit your revision, log on to https://www.editorialmanager.com/pone/ and select the 'Submissions Needing Revision' folder to locate your manuscript file.

Please include the following items when submitting your revised manuscript:

- A letter that responds to each point raised by the academic editor and reviewer(s). You should upload this letter as a separate file labeled 'Response to Reviewers'.
- A marked-up copy of your manuscript that highlights changes made to the original version. You should upload this as a separate file labeled 'Revised Manuscript with Track Changes'.
- An unmarked version of your revised paper without tracked changes. You should upload this as a separate file labeled 'Manuscript'.

If you would like to make changes to your financial disclosure, please include your updated statement in your cover letter. Guidelines for resubmitting your figure files are available below the reviewer comments at the end of this letter.

If applicable, we recommend that you deposit your laboratory protocols in protocols.io to enhance the reproducibility of your results. Protocols.io assigns your protocol its own identifier (DOI) so that it can be cited independently in the future. For instructions see: https://journals.plos.org/plosone/s/submission-guidelines#loc-laboratory-protocols. Additionally, PLOS ONE offers an option for publishing peer-reviewed Lab Protocol articles, which describe protocols hosted on protocols.io. Read more information on sharing protocols at https://plos.org/protocols?utm_medium=editorial-email&utm_source=authorletters&utm_campaign=protocols.

As the corresponding author, your ORCID iD is verified in the submission system and will appear in the published article. PLOS supports the use of ORCID, and we encourage all coauthors to register for an ORCID iD and use it as well. Please encourage your coauthors to verify their ORCID iD within the submission system before final acceptance, as unverified ORCID iDs will not appear in the published article. *Only*   the individual author can complete the verification step; PLOS staff *cannot*   verify ORCID iDs on behalf of authors.

We look forward to receiving your revised manuscript.

Kind regards,

Marwan Salih Al-Nimer, MD, PhD

Academic Editor

PLOS One

Journal Requirements:

When submitting your revision, we need you to address these additional requirements.

1. Please ensure that your manuscript meets PLOS ONE's style requirements, including those for file naming. The PLOS ONE style templates can be found at

https://journals.plos.org/plosone/s/file?id=wjVg/PLOSOne_formatting_sample_main_body.pdf and

https://journals.plos.org/plosone/s/file?id=ba62/PLOSOne_formatting_sample_title_authors_affiliations.pdf

2. In the online submission form, you indicated that “The datasets used and/or analysed during the current study are available from the corresponding author on reasonable request.”

All PLOS journals now require all data underlying the findings described in their manuscript to be freely available to other researchers, either 1. In a public repository, 2. Within the manuscript itself, or 3. Uploaded as supplementary information.

This policy applies to all data except where public deposition would breach compliance with the protocol approved by your research ethics board. If your data cannot be made publicly available for ethical or legal reasons (e.g., public availability would compromise patient privacy), please explain your reasons on resubmission and your exemption request will be escalated for approval.

3. PLOS requires an ORCID iD for the corresponding author in Editorial Manager on papers submitted after December 6th, 2016. Please ensure that you have an ORCID iD and that it is validated in Editorial Manager. To do this, go to ‘Update my Information’ (in the upper left-hand corner of the main menu), and click on the Fetch/Validate link next to the ORCID field. This will take you to the ORCID site and allow you to create a new iD or authenticate a pre-existing iD in Editorial Manager.

4. Please ensure that you refer to Figure 5 in your text as, if accepted, production will need this reference to link the reader to the figure.

5. Please upload a copy of Figure 5, to which you refer in your text on page xx. If the figure is no longer to be included as part of the submission please remove all reference to it within the text.

6. Please include your tables as part of your main manuscript and remove the individual files. Please note that supplementary tables (should remain/ be uploaded) as separate "supporting information" files

7. Please include captions for your Supporting Information files at the end of your manuscript, and update any in-text citations to match accordingly. Please see our Supporting Information guidelines for more information: http://journals.plos.org/plosone/s/supporting-information.

8. If the reviewer comments include a recommendation to cite specific previously published works, please review and evaluate these publications to determine whether they are relevant and should be cited. There is no requirement to cite these works unless the editor has indicated otherwise.

[Note: HTML markup is below. Please do not edit.]

Reviewers' comments:

Reviewer's Responses to Questions

**Comments to the Author**

1. Is the manuscript technically sound, and do the data support the conclusions?

The manuscript must describe a technically sound piece of scientific research with data that supports the conclusions. Experiments must have been conducted rigorously, with appropriate controls, replication, and sample sizes. The conclusions must be drawn appropriately based on the data presented.

Reviewer #1: Yes

Reviewer #2: Partly

**********

2. Has the statistical analysis been performed appropriately and rigorously?

Reviewer #1: No

Reviewer #2: Yes

**********

3. Have the authors made all data underlying the findings in their manuscript fully available?

The PLOS Data policy requires authors to make all data underlying the findings described in their manuscript fully available without restriction, with rare exception (please refer to the Data Availability Statement in the manuscript PDF file). The data should be provided as part of the manuscript or its supporting information, or deposited to a public repository. For example, in addition to summary statistics, the data points behind means, medians and variance measures should be available. If there are restrictions on publicly sharing data—e.g. participant privacy or use of data from a third party—those must be specified.

Reviewer #1: Yes

Reviewer #2: No

**********

4. Is the manuscript presented in an intelligible fashion and written in standard English?

PLOS ONE does not copyedit accepted manuscripts, so the language in submitted articles must be clear, correct, and unambiguous. Any typographical or grammatical errors should be corrected at revision, so please note any specific errors here.

Reviewer #1: Yes

Reviewer #2: Yes

**********

5. Review Comments to the Author

Please use the space provided to explain your answers to the questions above. You may also include additional comments for the author, including concerns about dual publication, research ethics, or publication ethics. (Please upload your review as an attachment if it exceeds 20,000 characters)

Reviewer #1: Title: Effectiveness of Exercise-Based Rehabilitation for Depressive Symptoms, Anxiety, and Health-Related Quality of Life in Adults With Rheumatoid Arthritis: A Systematic Review and Meta-Analysis of Randomized Controlled Trials

General:

This is an interesting and clinically relevant study addressing rheumatoid arthritis (RA), a condition well known not only for joint pain and functional limitation, but also for its substantial psychological burden, including depression, anxiety, and impaired health-related quality of life (HRQoL). The topic is important because many patients with RA are often reluctant to engage in exercise due to fear that physical activity may worsen pain or joint damage. The authors appropriately highlighted that evidence regarding exercise as an adjunctive therapy for psychological outcomes in RA remains heterogeneous and inconsistent, which provides a strong rationale for conducting this systematic review and meta-analysis.

Several methodological and reporting issues should be clarified and strengthened.

Introduction:

1. The introduction is relatively lengthy and somewhat repetitive in several sections.

2. Some theoretical explanations are overly extensive and delay the presentation of the primary research objective.

3. It remains unclear whether the authors intended to evaluate exercise interventions broadly or compare the effectiveness of different exercise modalities.

Suggestion: The authors may consider discussing fear-avoidance behavior or kinesiophobia in RA patients, as this is highly relevant to exercise adherence and rehabilitation participation.

Methods:

1. The manuscript should more explicitly emphasize adherence to the PRISMA 2020 guideline. Although PRISMA was mentioned, this reporting framework should be highlighted more prominently as the methodological standard.

2. The inclusion criteria regarding exercise interventions were very broad.

The review combined aerobic exercise, resistance training, walking programs, yoga, Tai Chi, and Yi Jin Jing into pooled analyses, which may substantially increase clinical heterogeneity.

3. The exclusion criteria were insufficiently described.

The manuscript only briefly mentioned exclusion of mixed inflammatory populations when RA data were unavailable separately. It remains unclear: whether non-English studies were excluded, whether grey literature was included, whether conference abstracts were considered, whether patients with severe psychiatric disorders were excluded, or whether co-interventions were allowed.

4. The manuscript did not sufficiently explain how different psychological instruments were harmonized beyond the use of standardized mean differences.

5. Publication bias and small-study effects were only briefly discussed.

Suggestion:

Additional subgroup analyses based on: exercise intensity, supervision, intervention duration, disease activity, baseline depression severity,

may improve clinical interpretability.

Results:

1. Clinical heterogeneity was substantial regarding: exercise modality, intervention duration, frequency, comparator groups, outcome instruments.

2. Most outcomes relied on self-reported questionnaires, increasing susceptibility to expectancy bias.

3. Several included studies had relatively small sample sizes (<60 participants).

4. Minimal clinically important differences (MCID) were not discussed.

Suggestion: Consider adding a dedicated table summarizing FITT principles (frequency, intensity, time, type), additional analyses comparing supervised versus home-based exercise may be clinically valuable, reporting absolute effects alongside SMDs may improve interpretability for clinicians.

Discussion:

1. The discussion is overly long and occasionally repetitive.

2. Some mechanistic explanations are speculative and excessively broad.

3. Certain sections resemble a narrative review rather than a focused interpretation of study findings.

Conclusion:

1. Practical recommendations for clinicians remain somewhat limited.

Final Recommendation

This manuscript addresses a clinically important and underrecognized aspect of RA management, namely the psychological impact of the disease and the potential role of exercise-based rehabilitation. The systematic review and meta-analysis are generally well conducted and aligned with PRISMA methodology. However, several methodological clarifications and improvements are needed, particularly regarding heterogeneity, exclusion criteria, and interpretation of clinical significance, before publication.

Reviewer #2: The study aimed to investigate the effects of structured exercise interventions with depressive symptoms, anxiety, and HRQoL in adults with RA, as well as to explore whether the effects differed between conventional exercise and mind-body exercise.

The manuscript is well delimited and follows the available reporting guidelines. The introduction is well grounded and appropriately contextualizes the research problem. In addition, the discussion and conclusion are consistent with and limited to the findings presented in the results.

However, the absence of the supplementary files (eTables) prevented a proper assessment of whether each methodological step was rigorously conducted, as well as verification of the information reported in the manuscript. I am unsure whether this omission was due to an issue on the part of the journal or the authors.

Additionally, instead of using the ROB tool, the authors could consider adopting the RoB 2 tool, which may provide a more robust assessment of risk of bias for randomized studies.

Finally, given the heterogeneity of the interventions included, I suggest that the authors consider the possibility of conducting a network meta-analysis.

**********

6. PLOS authors have the option to publish the peer review history of their article (what does this mean?). If published, this will include your full peer review and any attached files.

If you choose “no”, your identity will remain anonymous but your review may still be made public.

**Do you want your identity to be public for this peer review?**   For information about this choice, including consent withdrawal, please see our Privacy Policy.

Reviewer #1: No

Reviewer #2: **Yes:**  Danilo Reis Coimbra

**********

[NOTE: If reviewer comments were submitted as an attachment file, they will be attached to this email and accessible via the submission site. Please log into your account, locate the manuscript record, and check for the action link "View Attachments". If this link does not appear, there are no attachment files.]

To ensure your figures meet our technical requirements, please review our figure guidelines: https://journals.plos.org/plosone/s/figures

You may also use PLOS’s free figure tool, NAAS, to help you prepare publication quality figures: https://journals.plos.org/plosone/s/figures#loc-tools-for-figure-preparation.

NAAS will assess whether your figures meet our technical requirements by comparing each figure against our figure specifications.

# Author comment: Author response to Decision Letter 1

**DOI:** 10.1371/journal.pone.0352173.r002

4 Jun 2026

Dear Editor,

Thank you very much for the opportunity to submit our revised manuscript. We sincerely appreciate the careful evaluation of our manuscript by the Editorial Office and both reviewers, as well as the valuable and constructive comments provided. In response to the Editorial Office requirements and the reviewers’ suggestions, we have carefully revised the manuscript and addressed each comment point by point. A detailed point-by-point response is provided in the attached Response to Reviewers document.

In this revised submission, we have made every effort to improve the manuscript and the related submission files, including the manuscript text, figures, tables, Supporting Information files, Data Availability Statement, ORCID validation, and figure citations. All figures have been revised according to the PLOS ONE figure guidelines and checked using the NAAS tool. We have also submitted both the marked-up revised manuscript and the clean revised manuscript for your review.

We sincerely hope that the revised manuscript and our point-by-point responses have adequately addressed the concerns raised by the Editorial Office and the reviewers. If any further revisions are needed, we would be very willing to make additional changes according to your guidance.

Thank you again for your time, consideration, and valuable help.

Sincerely,

Yan Xiao

On behalf of all authors

Attachment Submitted filename: Response Letter .docx

# Editor report: Decision Letter 1

**DOI:** 10.1371/journal.pone.0352173.r003

**Authors:** Marwan Salih Al-Nimer

8 Jun 2026

Effectiveness of exercise-based rehabilitation for depressive symptoms, anxiety, and health-related quality of life in adults with rheumatoid arthritis: A systematic review and meta-analysis of randomized controlled trials

PONE-D-26-20182R1

Dear Dr. yan xiao,

We’re pleased to inform you that your manuscript has been judged scientifically suitable for publication and will be formally accepted for publication once it meets all outstanding technical requirements.

Within one week, you’ll receive an e-mail detailing the required amendments. When these have been addressed, you’ll receive a formal acceptance letter and your manuscript will be scheduled for publication.

An invoice will be generated when your article is formally accepted. Please note, if your institution has a publishing partnership with PLOS and your article meets the relevant criteria, all or part of your publication costs will be covered. Please make sure your user information is up-to-date by logging into Editorial Manager at Editorial Manager® and clicking the ‘Update My Information' link at the top of the page. For questions related to billing, please contact billing support.

If your institution or institutions have a press office, please notify them about your upcoming paper to help maximize its impact. If they’ll be preparing press materials, please inform our press team as soon as possible -- no later than 48 hours after receiving the formal acceptance. Your manuscript will remain under strict press embargo until 2 pm Eastern Time on the date of publication. For more information, please contact onepress@plos.org.

Kind regards,

Marwan Salih Al-Nimer, MD, PhD

Academic Editor

PLOS One

Additional Editor Comments (optional):

Reviewers' comments:

# Editor report: Acceptance letter

**DOI:** 10.1371/journal.pone.0352173.r004

**Authors:** Marwan Salih Al-Nimer

PONE-D-26-20182R1

PLOS One

Dear Dr. Yang,

I'm pleased to inform you that your manuscript has been deemed suitable for publication in PLOS One. Congratulations! Your manuscript is now being handed over to our production team.

At this stage, our production department will prepare your paper for publication. This includes ensuring the following:

* All references, tables, and figures are properly cited

* All relevant supporting information is included in the manuscript submission,

* There are no issues that prevent the paper from being properly typeset

You will receive further instructions from the production team, including instructions on how to review your proof when it is ready. Please keep in mind that we are working through a large volume of accepted articles, so please give us a few days to review your paper and let you know the next and final steps.

Lastly, if your institution or institutions have a press office, please let them know about your upcoming paper now to help maximize its impact. If they'll be preparing press materials, please inform our press team within the next 48 hours. Your manuscript will remain under strict press embargo until 2 pm Eastern Time on the date of publication. For more information, please contact onepress@plos.org.

You will receive an invoice from PLOS for your publication fee after your manuscript has reached the completed accept phase. If you receive an email requesting payment before acceptance or for any other service, this may be a phishing scheme. Learn how to identify phishing emails and protect your accounts at https://explore.plos.org/phishing.

If we can help with anything else, please email us at customercare@plos.org.

Thank you for submitting your work to PLOS ONE and supporting open access.

Kind regards,

PLOS ONE Editorial Office Staff

on behalf of

Professor Marwan Salih Al-Nimer

Academic Editor

PLOS One
